# Effective and safe proton pump inhibitor therapy in acid-related diseases – A position paper addressing benefits and potential harms of acid suppression

**DOI:** 10.1186/s12916-016-0718-z

**Published:** 2016-11-09

**Authors:** Carmelo Scarpignato, Luigi Gatta, Angelo Zullo, Corrado Blandizzi, for the SIF-AIGO-FIMMG Group, Carmelo Scarpignato, Corrado Blandizzi, Luigi Gatta, Angelo Zullo, Anna Kohn, Gioacchino Leandro, Antonio Balzano, Alberto Chiriatti, Walter Marocco, on behalf of the Italian Society of Pharmacology, the Italian Association of Hospital Gastroenterologists, and the Italian Federation of General Practitioners

**Affiliations:** 1 Clinical Pharmacology & Digestive Pathophysiology Unit, Department of Clinical & Experimental Medicine, University of Parma, Maggiore University Hospital, Cattani Pavillon, I-43125 Parma, Italy; 2 Gastroenterology & Endoscopy Unit, Versilia Hospital, Azienda USL Toscana Nord Ovest, Lido di Camaiore, Italy; 3 Division of Gastroenterology & Digestive Endoscopy, Nuovo Regina Elena Hospital, Rome, Italy; 4 Division of Pharmacology, Department of Clinical & Experimental Medicine, University of Pisa, Pisa, Italy

**Keywords:** Proton pump inhibitors, Antisecretory drugs, Efficacy, Safety, Appropriateness, Acid-related diseases

## Abstract

**Background:**

The introduction of proton pump inhibitors (PPIs) into clinical practice has revolutionized the management of acid-related diseases. Studies in primary care and emergency settings suggest that PPIs are frequently prescribed for inappropriate indications or for indications where their use offers little benefit. Inappropriate PPI use is a matter of great concern, especially in the elderly, who are often affected by multiple comorbidities and are taking multiple medications, and are thus at an increased risk of long-term PPI-related adverse outcomes as well as drug-to-drug interactions. Herein, we aim to review the current literature on PPI use and develop a position paper addressing the benefits and potential harms of acid suppression with the purpose of providing evidence-based guidelines on the appropriate use of these medications.

**Methods:**

The topics, identified by a Scientific Committee, were assigned to experts selected by three Italian Scientific Societies, who independently performed a systematic search of the relevant literature using Medline/PubMed, Embase, and the Cochrane databases. Search outputs were distilled, paying more attention to systematic reviews and meta-analyses (where available) representing the best evidence. The draft prepared on each topic was circulated amongst all the members of the Scientific Committee. Each expert then provided her/his input to the writing, suggesting changes and the inclusion of new material and/or additional relevant references. The global recommendations were then thoroughly discussed in a specific meeting, refined with regard to both content and wording, and approved to obtain a summary of current evidence.

**Results:**

Twenty-five years after their introduction into clinical practice, PPIs remain the mainstay of the treatment of acid-related diseases, where their use in gastroesophageal reflux disease, eosinophilic esophagitis, *Helicobacter pylori* infection, peptic ulcer disease and bleeding as well as, and Zollinger–Ellison syndrome is appropriate. Prevention of gastroduodenal mucosal lesions (and symptoms) in patients taking non-steroidal anti-inflammatory drugs (NSAIDs) or antiplatelet therapies and carrying gastrointestinal risk factors also represents an appropriate indication. On the contrary, steroid use does not need any gastroprotection, unless combined with NSAID therapy. In dyspeptic patients with persisting symptoms, despite successful *H. pylori* eradication, short-term PPI treatment could be attempted. Finally, addition of PPIs to pancreatic enzyme replacement therapy in patients with refractory steatorrhea may be worthwhile.

**Conclusions:**

Overall, PPIs are irreplaceable drugs in the management of acid-related diseases. However, PPI treatment, as any kind of drug therapy, is not without risk of adverse effects. The overall benefits of therapy and improvement in quality of life significantly outweigh potential harms in most patients, but those without clear clinical indication are only exposed to the risks of PPI prescription. Adhering with evidence-based guidelines represents the only rational approach to effective and safe PPI therapy.

Please see related Commentary: doi:10.1186/s12916-016-0724-1.

## Background

The introduction of proton pump inhibitors (PPIs) into clinical practice has revolutionized the management of acid-related diseases. Pharmacological acid suppression has been so successful in healing peptic ulcer (PU) and managing patients with gastroesophageal reflux disease (GERD) that elective surgery for ulcer disease has been virtually abolished and anti-reflux operations are today performed only in selected patients. Along the same lines, the incidence of non-steroidal anti-inflammatory drug (NSAID)-associated gastropathy has largely been reduced, despite the increased use of these medications in the aging population [1].

Despite the fact that PPIs are far from being the ideal antisecretory drugs [2] and that new longer-acting compounds with extended acid suppression are being developed [3–5], they remain, no doubt, the most effective currently available medications and are widely prescribed in all age populations. Healthcare providers are increasingly prescribing PPIs for prolonged, sometimes lifetime, use and there is growing concern for the potential adverse effects resulting from such long-term therapy [6, 7]. Soon after the introduction of omeprazole, the first PPI, into the market, Jean Paul Galmiche, a leading French gastroenterologist, wrote a thoughtful article [8] anticipating that the unprecedented clinical efficacy of these drugs would have lead (patients and physicians alike) to addiction, and indeed, this is the case. Once on a PPI, the majority of patients stay on long-term PPIs, often indefinitely [9], especially the elderly [10].

Studies in primary care and emergency settings suggest that PPIs are frequently prescribed for inappropriate indications or for indications where their use offers little benefit [11]. Patients admitted to hospital frequently are started on PPIs, often inappropriately [12], and these medications are continued, following discharge, by primary care physicians. Indeed, inadequate recommendations for PPIs in discharge letters are quite frequent [13]. This prescription habit may lead to a continuation of PPI therapy in primary care, thereby unnecessarily increasing polypharmacy and the risk of adverse events as well as burdening the public health budget. In this connection, an Italian study [14] found that the persistence rate of PPI therapy is fairly high, after both appropriate and inappropriate prescriptions (62 % and 71 %, respectively). The general practitioners’ attitude to continuing or discontinuing PPIs depends on their level of knowledge and their perceptions of hospital physicians’ competence as well as the threshold to prescribing in hospitals [15].

The introduction of generic PPIs into the market has been followed by an increasing rate of PPI prescribing related to chronic treatments, unlicensed indications, and therapeutic substitutions [16]. Furthermore, since PPIs are now available over-the-counter [17], patients can have free access to them and for long periods of time, without seeking medical attention [18, 19]. Counseling is therefore important to ensure that patients understand that failure of symptoms to resolve or a rapid symptom relapse while taking a PPI is an indication to consult a physician. Furthermore, concerns about potential masking of a more serious pathology, such as malignancy, should not be overlooked [19].

Inappropriate PPI use is a matter of great concern, especially in the elderly, who are often affected by multiple comorbidities and are taking multiple medications, and are thus at an increased risk of long-term PPI-related adverse outcomes and drug-to-drug interactions (DDIs). There is indeed a strong relationship between the number of administered drugs and potential, clinically relevant DDIs [20], particularly in older adults [21]. As a consequence, the number of yearly papers reporting PPI-related adverse events and/or PPI-drug interactions has steadily increased over the past decade [22–25].

Together with inappropriate use, underuse is also matter of concern. For instance, despite all guidelines supporting the use of gastroprotection with PPIs in at-risk patients treated with NSAIDs [26–28], low prescription rates of gastroprotective medications have been reported, although these rates have increased progressively (for review see [28]). In Italy, three studies [29–31] reported a substantial misuse of gastroprotection in primary care. In particular, an underuse rate of 25 − 30 % or an overuse (young patients without any concomitant risk factor) as high as 57.5 % were observed. In addition, half PPI doses or ineffective H_2_-receptor antagonist (H_2_RA) treatment were prescribed in another 10 % of subjects [30].

According to the data provided by the Organization for Economic Cooperation and Development [32], PPI use is widespread and currently increasing, especially in some European countries. Prescription rates have risen substantially, not only as a consequence of the replacement of H_2_RAs but also because of an expansion in the overall market. PPI utilization does not appear to be commensurate with prevalence of acid-related disease (particularly with GERD and NSAID-gastropathy) nor with prescribing guidelines, thus leading to significant incremental costs to both patients and national health systems. Inappropriate prescribing is therefore high and costly. In a US teaching hospital, the estimated cost of inpatient and outpatient inappropriate use of PPIs was $12,272 and $59,272, respectively [33]. This trend is also being observed in most industrialized countries and Italy is no exception.

Taking all the above considerations into account, the Italian Society of Pharmacology (SIF), the Italian Association of Hospital Gastroenterologists (AIGO), and the Italian Federation of General Practitioners (FIMMG) considered it wise to review the current literature on PPI use and to prepare a position paper addressing the benefits and potential risks of acid suppression with the aims of providing evidence-based guidelines for appropriate use of these medications.

## Methods

The three scientific societies (SIF, AIGO, FIMMG), promoters of the endeavor, identified experts amongst their members in order to set-up the Scientific Committee, who defined the methodology to be followed in the preparation of the position paper.

The methodology adopted to process the recommendations consisted of four subsequent steps. In a meeting, held in Rome at the beginning of 2015, the Scientific Committee identified the following 13 clinically relevant areas on which primary care physicians and gastroenterologists are uncertain about how to prescribe PPIs in patients with acid-related diseases and where drug-misuse was found to be common:

1. GERD
2. Eosinophilic esophagitis (EoE)
3. *H. pylori* eradication and PU disease
4. Zollinger–Ellison syndrome (ZES)
5. Stress ulcer prophylaxis (SUP)
6. Dyspepsia
7. NSAID-associated gastrointestinal symptoms and lesions
8. Corticosteroid use
9. Antiplatelet or anticoagulant therapy
10. PU bleeding
11. Patients with cancer
12. Cirrhosis
13. Pancreatic disease

Since PPIs are often used long-term, the benefit-to-harm balance of such therapy was also addressed.

Each selected topic was assigned to a given expert, who carried out an independent systematic search of the relevant literature using Medline/PubMed, Embase, and the Cochrane databases. Search outputs were distilled, paying more attention to systematic reviews and meta-analyses (where available) representing the best evidence.

For each topic, a draft was prepared and circulated amongst all the members of the Scientific Committee. Each expert then provided her/his input to the writing, suggesting changes and the inclusion of new material and/or additional relevant references. Following preparation of the revised draft, each topic was addressed to the Core Writing Group (CS, LG, AZ, CB), who prepared the first draft of the full manuscript, which was examined in Bologna on June 2015. During the meeting, each single topic was thoroughly discussed and each statement concerning the summary of current evidence refined with regard to both content and wording.

The Core Writing Group then incorporated all the suggestions raised during the Bologna meeting and prepared the final draft. In doing so, an updated literature search was performed and the most recent evidence included. This revised document was then sent to Italian and International experts (*see Acknowledgements section*) for review. Any changes resulting from comments received by the external experts were made on the basis of scientific and editorial merit in order to produce the final version of the position paper.

## Results

### PPIs for GERD

#### Summary of the current evidence

*PPIs represent the mainstay of medical treatment of esophageal manifestations of GERD; however, their benefits (if any) in extra-digestive GERD are still uncertain. Eight-week therapy with standard (once daily) dose PPIs can achieve healing of reflux esophagitis and symptom relief in more than 80 % of patients with typical symptoms. When a functional investigation is added to a negative endoscopy in making the diagnosis, PPI efficacy in GERD and non-erosive reflux disease (NERD) appears comparable. Being a chronic, relapsing disease, GERD (as well as NERD) requires long-term PPI treatment, which can be continuous, intermittent or on-demand. Profound and individually tailored maximal acid suppression is needed in patients with Barrett’s esophagus not only to control gastroesophageal reflux (GER) but also in the hope to achieve a chemopreventive effect against neoplastic transformation.*

GER (i.e., the reflux of gastric contents into the esophagus) is a physiological phenomenon, occurring in everybody, especially after large and fat meals. Under physiological conditions, efficient esophageal clearing mechanisms return most of the refluxed material to the stomach and symptoms do not occur [34]. However, when the reflux of gastric contents is large or aggressive enough, it causes troublesome symptoms and/or complications and adversely affects health-related quality of life, giving rise to GERD [35]. The Montreal consensus subclassified the disease into esophageal and extra-esophageal syndromes, with established or proposed associations with GER [36]. Up to two-third of patients with esophageal symptoms have a macroscopically normal mucosa at endoscopy. Such patients are usually considered to have NERD [37, 38].

GERD is primarily a motor disorder and its pathogenesis is multifactorial. The main motility abnormalities include an impaired function of the lower esophageal sphincter, an abnormal esophageal clearance and a delayed gastric emptying in up to 40 % of cases. The presence of hiatal hernia favors reflux, but this association is not mandatory. The ultimate consequence of the above motor abnormalities is the presence of acid in the wrong place (i.e., in contact with the esophageal mucosa) [39]. In addition, the amount of reflux increases markedly after meals in both healthy subjects and in GERD patients, an event almost exclusively due to the increase of transient (inappropriate) lower esophageal sphincter relaxations by food-induced gastric accommodation. Despite the buffering content of food, the pH of the material refluxed into the distal esophagus is acidic due to the presence of an “acid pocket”, which occurs in both healthy subjects and GERD patients. It represents an area of unbuffered gastric acid that accumulates in the proximal stomach after meals and serves as a reservoir for acid reflux [40]. The abnormal esophageal exposure to acid, on the other hand, is not secondary to gastric acid hypersecretion, which has been documented in only a small subset of GERD patients [39]. All the above pathophysiological mechanisms are exaggerated in obese subjects [41, 42].

Since effective drugs capable of controlling the esophageal motor abnormalities are currently lacking, the mainstay of medical treatment for GERD are antisecretory drugs, which act indirectly by reducing the amount and concentration of gastric secretion available for reflux, thus lessening the aggressive power of the refluxed material [43]. PPIs also reduce the size of the acid pocket and increase the pH (from 1 to 4) of its content [40]. The clinical efficacy of these drugs has been clearly shown in many studies and the superiority of PPIs over H_2_RAs has been established beyond doubt [44]. The greater pharmacodynamic effect of PPIs depends on their ability to block the final step in the production of acid, regardless of the secretory stimulus. Moreover, PPIs are relatively more effective during the daytime than the nighttime and this leads to a better control of post-prandial reflux events [44].

Eight-week therapy with standard (once daily) dose PPIs can achieve healing of reflux esophagitis in more than 80 % of patients [45], a rate depending on the severity of mucosal lesions [46, 47]. This healing rate can be further improved by doubling the PPI dose [45]. Meta-analyses have shown that when compared to omeprazole, lansoprazole and pantoprazole, esomeprazole achieves the highest healing rates of reflux esophagitis in the short term [46, 48, 49]. The more favorable clinical benefit of esomeprazole appears negligible in less severe esophagitis (A & B according to the Los Angeles classification [50, 51]), but it might be important in more severe disease [48]. Vonoprazan, a member of the new generation reversible PPIs (called potassium-competitive acid blockers), is able to achieve higher intragastric pH, effectively controlling both daytime and nighttime acid secretion [5]. As a consequence, it proved to be capable of healing almost 100 % of severe (grades C and D) esophagitis [52], a benefit also maintained during the remission phase [53].

It is worth mentioning that currently available PPI regimens do not provide the same control of intragastric pH, evaluated both in terms of mean pH over the 24 hours and percentage of time spent at pH > 4. This has been repeatedly demonstrated in patients with GERD [54–56] or taking NSAIDs [57]. A large meta-analysis [58], including 57 studies measuring intragastric pH after different PPI regimens, found that the relative potencies of the five compounds, compared to omeprazole, were 0.23, 0.90, 1.60, and 1.82 for pantoprazole, lansoprazole, esomeprazole, and rabeprazole, respectively. This lack of pharmacodynamic equivalence should be taken into account when switching from a given PPI to another.

PPIs are effective in obtaining symptom relief in both erosive and non-erosive disease [59]. Their efficacy for the relief of regurgitation is, however, modest and considerably lower than that achieved for heartburn [60]. The myth that PPIs are less effective in NERD has recently been dispelled by a meta-analysis [61] showing that, when a functional investigation (pH-metry or pH-impedance recording) is added to a negative endoscopy to objectively confirm this condition, the estimated complete symptom response rate after PPI therapy is comparable to that observed in patients with erosive disease.

However, NERD is an umbrella term, including at least four different patient subgroups [38], of whom only those where acid is implicated in symptom generation (i.e., true NERD and patients with acid hypersensitive esophagus) are clearly responsive to PPIs [62]. This is not the case of patients who are hypersensitive to nonacidic reflux or those with functional heartburn. According to Rome IV criteria [63], both acid hypersensitive esophagus (now called reflux hypersensitivity) and functional heartburn are functional gastrointestinal (GI) disorders, which should no longer be included in GERD. The lack of abnormal acid exposure and symptom-reflux association makes patients with functional heartburn not responsive to PPIs. This subgroup of subjects may benefit from visceral analgesics (e.g., antidepressants) [64].

Although not as frequent as previously suggested, PPI-refractory heartburn, occurring more commonly in NERD than in erosive disease, does nevertheless exist. Some 20 % (range 15–27 %) of correctly diagnosed and appropriately treated patients do not respond to PPI therapy at standard doses [65]. To ascertain whether they are “truly” PPI-resistant, compliance and adherence to treatment should be checked. Indeed, PPIs are often taken inappropriately, with only 27 % of GERD patients dosing their PPI correctly and only 12 % dosing it optimally in a USA survey [66]. Although a standard PPI dose can occasionally control symptoms, nocturnal intragastric acidity often remains elevated (with nocturnal acid breakthrough, NAB) in these patients. A split regimen (either standard or double dose) of PPIs b.i.d. (before breakfast and before evening meal) provides superior acid control. In patients with persistent nocturnal symptoms, the addition of an H_2_RA at bedtime may be indicated to control NAB and associated esophageal acidification [3, 62, 67, 68], despite the likely development of tolerance to H_2_RAs [69]. The majority of patients, however, reported persistent improvement in GERD symptoms from night-time H_2_RA use [67]. To reduce the development of tolerance, on demand or cyclic dosing may be preferable, but this approach has not been specifically studied.

GERD and NERD are chronic, relapsing diseases. Six months after cessation of treatment, symptomatic relapse is rapid and frequent (i.e., in 90 % of endoscopy-positive and 75 % of endoscopy-negative patients [70]). PPIs, both at a full and half dose, are able to maintain patients in remission, with a superior efficacy of the full dose (NNT = 9.1) [71]. Esomeprazole 20 mg is the only step-down dose PPI able to maintain a significantly higher proportion of GERD patients in symptomatic remission, as compared to lansoprazole 15 mg [49, 72] or pantoprazole 20 mg [49].

Since PPIs do not correct the underlying pathophysiological motor abnormalities responsible for GERD, a continuous treatment is required to maintain all patients in remission. In the LOTUS trial [73], comparing long-term esomeprazole therapy with anti-reflux surgery (ARS), the estimated remission rate at 5 years was 92 %, higher than that reported with omeprazole in the SOPRAN study (57 %) [74]. However, while the PPI dose in the SOPRAN trial was fixed, in the LOTUS investigation, patients whose reflux symptoms were not adequately controlled by a standard maintenance regimen (i.e., esomeprazole, 20 mg/day) were allowed to increase the dosage to 40 mg once daily and then to 20 mg twice daily. This dose titration may have contributed to the improved remission rate and suggests that long-term maintenance therapy should be individualized. Indeed, the number and severity of relapses are highly variable amongst patients. Infrequent reflux symptoms are less likely to be chronic and may respond to different management strategies. There are basically three different long-term approaches for GERD treatment with PPIs: continuous (i.e., every day), intermittent (i.e., cycles of daily PPI administration), or on-demand (i.e., symptom-driven) therapy, each selected on the basis of patients’ clinical characteristics [75].

One third of patients, submitted to fundoplication, is reported to take acid-lowering compounds (mostly PPIs) after ARS, but only few studies have specified whether drug use was on a regular or occasional basis [76]. A meta-analysis of randomized controlled trials (RCTs) [77] found that – after ARS – 14 % of patients still require antisecretory drugs. This figure increases with the duration of follow-up and up to one third of patients required antisecretory drugs after 10 years. The data from non-randomized studies [78], which are higher than the estimation provided by randomized studies (i.e., 20 % of patients under acid suppression), are probably more representative of the current clinical practice.

Although medication use is often considered as an outcome measure for successful ARS, some studies have shown that antisecretory drug use does not correlate with true recurrent reflux in most patients [76] and does not necessarily indicate a failure of the procedure. A significant proportion of patients taking medications after operation are using them to relieve non-reflux symptoms and only one third of patients displays an abnormal esophageal exposure to acid after surgery [76]. Therefore, many patients take PPIs despite the lack of objective evidence of GERD on esophageal testing. The causes of persistent symptoms after surgery remain unclear. Non-GERD symptoms might be due to increased esophageal sensitivity while other symptoms (like bloating, early satiety and nausea) may be unmasked when reflux symptoms improve [79–81]. A careful selection of patients and thorough follow-up is needed to avoid unnecessary acid suppression in post-surgical patients.

Before embarking on long-term treatment, an attempt to stop acid suppression must always be considered. Of the various interventions (patient’s education, life-style modifications, abrupt withdrawal, and tapering), tapering is the more effective discontinuation strategy [82]. Abrupt withdrawal might be followed by rebound acid hypersecretion and exacerbation of symptoms [83]. Weight loss appears to be another strategy in obese/overweight patients. Indeed, in one study, up to 54 % of subjects compliant to a hypocaloric diet were able to stop PPI therapy, with an additional 32 % being able to halve the dose [84]. All the above attempts should be considered also in patients who are already on long-term acid suppression.

Continuous maintenance therapy is indicated in patients with Barrett’s esophagus of any mucosal length, owing to the potential chemopreventive activity of PPIs against neoplastic transformation, a property advocated by the American College of Gastroenterology (ACG) [85] and American Gastroenterological Association [86], but denied by the British Society of Gastroenterology guidelines [87]. Indeed, a recent meta-analysis of observational studies showed that PPI use is associated with a 71 % reduction in risk of esophageal adenocarcinoma and/or high-grade dysplasia in this patient population (adjusted odds ratio (OR) = 0.29) [88]. Despite a contrary opinion of the American Gastroenterological Association [86], current evidence suggests that standard PPI therapy is unable to normalize esophageal exposure to acid in the vast majority of patients with Barrett’s esophagus. Profound and individually tailored maximal acid suppression is needed not only to control GER, but also in the hope to achieve a better chemopreventive effect [89].

In all those patients with GERD requiring long-term PPI therapy, *H. pylori* should be sought and – if present – eradicated, particularly in young patients. This approach, recommended by international guidelines [90, 91], is needed to prevent the development of atrophic gastritis or worsening of any preexisting one, with potential for neoplastic transformation [92]. However, in accordance with the Food and Drug Administration, ACG guidelines [93] do not recommend routine screening for or treatment of *H. pylori* infection in GERD patients (strong recommendation, low level of evidence).

Conversely from typical symptoms, the efficacy of PPIs on extra-esophageal manifestations of GERD is uncertain. This uncertainty could result, at least in part, from the available studies, which are not homogenous, with differences in patient selection, end-point considered, drug used, and regimen adopted. In addition, since extra-digestive symptoms may need higher PPI dose and clinical improvement may take a longer time to occur, only properly designed trials would be able to unravel a clinical response. Unfortunately, however, this has not always been the case.

The efficacy of PPIs in non-cardiac chest pain and extra-digestive GERD is disappointing. In these clinical conditions, PPIs are usually given twice daily and for extended periods (i.e., 3 or more months). However, evidence is often lacking and, where available, not strong enough to allow clear recommendations to be made.

With GERD being the most common and best-studied cause of non-cardiac chest pain, acid suppression is the initial pharmacological approach in this patient population. A systematic review showed that patients with endoscopic or pH-monitoring evidence of GERD tend to improve, but not resolve, with PPI therapy, whereas GERD-negative patients display little or no response [60], a result confirmed by a more recent meta-analysis [94]. PPIs might also improve symptoms related to atrial fibrillation and other supraventricular arrhythmias, especially after meals, in patients with proven GERD [95].

Despite the negative conclusions of a Cochrane meta-analysis [96], a recent review [97] suggests that a therapeutic benefit for acid-suppressive therapy in patients with chronic cough cannot be dismissed, advocating a rigorous patient selection that could allow the identification of patient subgroups likely to be responsive. On the contrary, no systematic reviews and meta-analyses [98–103] found any significant clinical benefit of PPI therapy over placebo in reflux laryngitis.

Asthma and GERD can often coexist, with reflux disease being reported in 40–80 % of patients with asthma. While asthma medications can trigger GERD [104, 105], PPIs might, on the contrary, improve asthma control. Here, again, an early Cochrane review [106] showed no benefit of PPI therapy on nocturnal symptom score and lung function, but a recent meta-analysis [96] – by selecting the morning peak expiratory flow rate as the primary outcome – disclosed a benefit of PPIs over placebo, which was greater in patients with proven GERD.

Despite the widespread use of PPIs in dental practice to manage the oral manifestations of GERD [107], treatment of dental erosions represents the only *objectively* documented clinical use [108].

In summary, while PPIs are the mainstay of medical treatment for esophageal manifestations of GERD, their benefit in extra-digestive GERD remains uncertain. The complexity of patient presentation is matched by the challenge in appropriate diagnosis of reflux as the cause for patients’ symptoms, which may also be related to other co-morbidities. Upper GI endoscopy and pH monitoring suffer from poor sensitivity, while laryngoscopy suffers from poor specificity in diagnosing reflux in this group of patients [109]. An empiric trial of PPIs could be the initial approach to diagnose and treat the potential underlying cause of these extra-esophageal symptoms. For those who improve with PPIs, GERD is presumed to be the etiology, but for those who do not respond, diagnostic testing with impedance and/or pH monitoring are reasonable to exclude continued acid or weakly acid reflux. In such cases, etiologies other than GERD may be pursued [109]. Difficult patients are best investigated and treated in referral centers.

### PPIs for eosinophilic esophagitis (EoE)

#### Summary of the current evidence

*PPIs are considered a first-line treatment in EoE. Other effective alternatives, such as dietary or topical corticosteroid therapy, should be used as second-line strategies, owing to long-term safety concerns (topical steroid therapy) and impairment of quality of life and nutritional inadequacy (dietary interventions). However, few data exist to guide specific recommendations on dose and duration of PPI initial therapy.*

EoE is a chronic immune-mediated inflammatory disorder, defined symptomatically by esophageal dysfunction and histologically by esophageal eosinophil-predominant inflammation [110, 111]. Originally thought to be a rare disease, its prevalence has greatly increased over the past 25 years. The allergic basis of EoE is supported by studies demonstrating that the underlying etiology for EoE is likely an aberrant “antigenic” or “immune” response, associated with consistent clinical and histologic abnormalities [112], and by their disappearance after antigen-free amino acid-based elemental diet [113].

It is important to emphasize that esophageal eosinophilia is a histological finding that requires interpretation in the clinical context and that esophageal eosinophilia alone does not define EoE; indeed, many other diseases have been associated with this histologic finding [114]. Although the presence of esophageal eosinophilia was first described in a subset of patients with GERD, it usually locates in the distal esophagus and never reaches the high density commonly observed in EoE, where it is associated with esophageal motor dysfunction, particularly dysphagia and food impaction [110, 111].

According to the ACG guidelines [115], PPI responsive esophageal eosinophilia (PPI-REE) should be diagnosed when patients have esophageal symptoms and histologic findings of esophageal eosinophilia, but demonstrate symptomatic and histologic response to PPIs. Originally categorized as a distinct clinical entity, PPI-REE is now considered a phenotype of EoE that is responsive to PPI therapy [110]. PPIs, therefore, no longer represent a diagnostic tool to distinguish between these two entities characterized by esophageal eosinophilia and symptoms, but a therapeutic option to be offered to patients with EoE. In this clinical setting, pH monitoring does not accurately predict response to PPI therapy [115], and similar remission rates have been documented in patients with both normal and pathologic pH monitoring [116]. In addition, symptom improvement is common with PPI therapy despite persistent eosinophilic infiltration [117].

A very recent meta-analysis [116], including 33 studies and 619 patients with EoE, found that PPI therapy led to a clinical response in 60.8 % and histologic remission in 50.5 % of patients. However, few data exist to guide specific recommendations on dosage and duration of PPI initial therapy. Retrospective data support the use of either once or twice daily use, but many of the PPI-REE studies used twice daily PPI dosing in the 20–40 mg range of the several available PPIs [115], for which there is a trend (albeit non-significant) towards an increased effectiveness [116].

The reason for PPI responsiveness of this condition is not completely understood but could be due to other, non-antisecretory effects of PPIs [118], of which the anti-inflammatory action [119] is the most relevant one. In vitro and in vivo studies suggest that the anti-inflammatory effects of PPI therapy rather than acid suppression alone may be responsible for this improvement through inhibition of the Th2-allergic pathway [120]. Indeed, like topical corticosteroids, PPIs down-regulated cytokine expression [121]. Alternatively, the dilated intercellular spaces and consequent increased mucosal permeability, present in EoE [122], may allow allergen penetration, which triggers subsequent recruitment of eosinophils to the esophageal epithelium. Some studies have shown that dilated intercellular spaces could recover after short-term PPI treatment in GERD (for review see [123]), a finding recently reported in patients with PPI-REE [124].

In summary, due to their safety profile, ease of administration, and high response rates (up to 60 % clinically), PPIs can be considered a first-line treatment for EoE. Recent data show that patients with EoE, responsive to topical steroids and diet, also respond to PPI treatment [125, 126]. However, the former approaches might be set aside as second-line, owing to long-term safety concerns (topical corticosteroid therapy) and impairment of quality of life and/or nutritional inadequacy (dietary interventions) [115, 116, 127].

### PPIs for *H. pylori* eradication and peptic ulcer (PU) disease

#### Summary of the current evidence

*PPIs represent a key component of any currently adopted regimens for* H. *pylori eradication. The degree and duration of acid suppression influence the eradication rate. While almost all* H. pylori-*positive ulcers are cured by* H. pylori eradication, H. pylori-*negative and NSAID/aspirin-negative PUs need high dose PPIs to be healed and, often, lifelong acid suppression is required to prevent recurrence.*

After the discovery by Warren and Marshall of the infectious etiology of PU disease in 1984 [128, 129], several lines of evidence confirmed that *H. pylori* eradication cures PU disease without the need for subsequent long-term maintenance antisecretory therapy [130–133], and can also be beneficial to other *H. pylori*-related diseases [90]. While previously used only to heal PUs, PPIs have become a key component of all the currently adopted eradication regimens [134], thus gaining a new role in the management of PU disease.

*H. pylori* is located within the gastric mucus layer, deep within the mucus-secreting glands of the antrum, attached to cells and even within cells, and is able to survive over a wide pH spectrum [134]. Since the survival capabilities of *H. pylori* within the stomach make its eradication difficult, several different drug combinations have been developed, with variable and inconsistent success, with no single therapy being effective worldwide. Therefore, the search for a new regimen to treat *H. pylori* infection still continues today [135].

An effective therapy should be able to eradicate the organism from each of these potential niches, which is an overwhelming task for any single antibiotic, whose in vitro susceptibility does not necessarily correlate with successful treatment in vivo. Since the very beginning, it was recognized that therapy with a single antibiotic leads to poor cure rates and various recipes were attempted, resulting in several effective combinations of antimicrobials, bismuth, and antisecretory drugs [136].

PPIs display several pharmacological actions that give them a place in the eradication regimens, namely:

1. They exert a direct antibacterial action against *H. pylori* [137, 138];
2. By increasing intra-gastric pH, they allow the microorganism to reach the growth phase and become more sensitive to antibiotics such as amoxicillin and clarithromycin [139];
3. They increase antibiotic stability [140] and efficacy [141];
4. By reducing gastric emptying [142] and mucus viscosity [143], they increase the gastric residence time and mucus penetration of antimicrobials.

The mechanisms underlying the antibacterial activity of PPIs are complex and have been detailed in a comprehensive review [134]. These compounds are able to bind *H. pylori* cells and the bactericidal activity correlates with the degree of binding. Electron microscopy studies revealed – after exposure to a PPI – a significant change in the morphology of the microorganism, with appearance of coccoid forms (known to be degenerating organisms) [134]. PPIs are potent inhibitors of *H. pylori* urease at all pH values. This effect translates into significant reduction of ammonia production. Since this bacterial metabolite is important for development of mucosal inflammation and subsequent mucosal ulceration [144], patients receiving PPIs in combination with antimicrobials may have the additional benefit of reducing one of the potent inflammatory stimuli.

The inhibitory activity on *H. pylori* urease has been confirmed in vivo. As a consequence, PPI administration results in the inability to detect the microorganism, thus interfering with the urease-based diagnostic tests [90, 145]. The temporary inability to detect the presence of *H. pylori* is termed suppression and simply reflects a decrease in the number of bacteria below the limits of detection [145]. It is worth mentioning that the infection affects the degree and the duration of acid inhibition achieved by antisecretory drugs. Amongst the mechanisms by which the microorganism could modify the pH-rising effect of PPIs, the buffering of *H. pylori*-generated ammonia on gastric acid remains the most convincing one [69].

A large clinical trial (the MACH-2 study) clearly showed that eradication rates achieved with two antibacterial agents (clarithromycin with either amoxicillin or metronidazole) are significantly lower than those achieved by the same two agents, given concomitantly with omeprazole [146]. These results were later confirmed by another RCT, where the combination of clarithromycin and tinidazole was evaluated with or without lansoprazole [147].

PPIs now represent the key component of any currently adopted regimens for *H. pylori* eradication, as recommended by Italian [148] and international guidelines [90, 149–151]. To be most effective, full dose PPIs should be given twice daily, concomitantly with antimicrobials as the mean intention-to-treat cure rates are greater in patients who use the high-dose PPI, compared with the standard-dose regimen [152, 153].

Eradication rates achieved with standard 1-week triple therapy (PPI-clarithromycin-amoxicillin) are dependent on CYP2C19 genotype [154]. Therefore, although any PPI can be selected, esomeprazole- and rabeprazole-based eradication regimens may show a better efficacy [155]. The biological plausibility of their superiority over other members of the PPI-class relies on their catabolism. Indeed, esomeprazole, the S-enantiomer of omeprazole, being - together with its metabolite (esomeprazole sulfone) - a powerful inhibitor of CYP2C19, does inhibit its own metabolism, rendering all subjects “slow metabolizers” [156]. This results in a more consistent acid suppression and might underline the slightly higher eradication rates reported with these PPIs [155]. Conversely from the other PPIs, the clearance of rabeprazole is much less dependent on CYP2C19 as it is predominantly metabolized non-enzymatically to rabeprazole thioether [157]. As a consequence, its antisecretory effect and the eradication rates of rabeprazole-based regimes are almost completely independent of genetic polymorphism [158]. As a matter of fact, a recent meta-analysis did show that, contrary to omeprazole and lansoprazole, the CYP2C19 genotype does not influence the eradication rate of esomeprazole- and rabeprazole-based therapies [159].

The importance of profound and long-lasting acid suppression for *H. pylori* eradication is illustrated by two studies showing a significantly higher intragastric pH and lower percent time spent at pH < 4 in patients successfully eradicated versus those who did not get rid of the bacterium [160, 161]. In addition, the eradication rate was higher in NAB-negative compared to NAB-positive patients [160]. Controlling intra-gastric acidity is therefore needed to achieve the best eradication rates [152, 162]. Indeed, some studies have shown that the use of high dose PPIs might result in high eradication rates even when one single antimicrobial agent is used [163–165]. The recent availability of more potent and longer-acting acid suppressants, namely vonoprazan [5], may facilitate the use of a dual therapy (i.e., acid suppressant plus amoxicillin) [166].

Although *H. pylori* infection remains the single most common cause of PUs, an increasing proportion of patients have *H. pylori*-negative ulcers [167]. The proportion is higher in the USA (and likely in Australia) than elsewhere, being only 4 % in Italy [168]. Although the precise etiology of these ulcers is unknown, some are caused by the use of aspirin or NSAIDs [169]. Indeed, together, *H. pylori* infection and NSAID use account for approximately 90 % of PU disease [170]. Patients with *H. pylori*-negative, NSAID/aspirin-negative (idiopathic) ulcers may have a more serious ulcer diathesis. While NSAID ulcers can be healed with PPIs (*see below*), idiopathic ulcers are likely to require long-term management with acid-suppressing drugs. PPIs are again the drugs of choice, although the optimal duration of treatment is undefined and might be lifelong [171].

### PPIs for Zollinger–Ellison syndrome (ZES)

#### Summary of the current evidence

*PPIs are the drugs of choice for the medical treatment of ZES, but relatively high doses (3–4 times the standard dose) are required compared with those used in other acid-related conditions. Patients with complicated ZES (severe GERD, Billroth II resections, and multiple endocrine neoplasia type 1 (MEN-1) with untreated hyperparathyroidism) are more difficult to treat and usually benefit from twice-a-day PPI dosing. The intravenous route may be required initially. When curative tumor removal is not possible, antisecretory therapy must be continued indefinitely. Interruption of PPI treatment can have detrimental consequences.*

ZES is a rare disorder characterized by the presence of a gastrin-producing tumor (gastrinoma), which leads to sustained hypersecretion of gastric acid and consequent PU disease (often with complications such as perforation, bleeding, etc.), diarrhea, or malabsorption. Gastrinomas usually develop in the non-beta islet cells of the pancreas or in the duodenal wall (40–90 %). Up to two-thirds are malignant. About 20–25 % of cases are seen in patients with MEN-1 syndrome [172, 173]. Nevertheless, even rarer, ZES symptoms may arise from a cholecystokinin-producing tumor [174], because both cholecystokinin (CCK) and gastrin are full agonists for the gastrin–CCK_2_ receptor of the parietal cell, whose stimulation drives gastric acid secretion.

Although somatostatin analogues (octreotide or lanreotide) could be used to reduce serum gastrin and gastric acid secretion [175–177], initial treatment is aimed at controlling the hypersecretion of gastric acid with an antisecretory drug. Dose titration using gastric acid analysis is the ideal way to best determine the lowest effective dose of medical therapy. Indeed, giving enough medication just to control symptoms is not considered adequate, and it is important that acid secretion is reduced below 10 mEq/h (or below 5 mEq/h in the post-surgical stomach) to avoid ulcer recurrence and complications [178]. According to available guidelines [176, 177], a PPI is the drug of choice, but high doses (3–4 times the standard dose, once daily) are required compared with those used in other acid-related disorders. Patients with complicated ZES (severe GERD, Billroth II resections, and MEN-1 with untreated hyperparathyroidism, etc.) are more difficult to treat and may require twice-a-day PPI dosing. The intravenous route (e.g., pantoprazole 80 mg every 8 hours [179]) may be required initially. Once symptoms have been controlled, the tumor can be investigated for surgical excision [178]. When curative removal is not possible, antisecretory therapy must be continued indefinitely. In patients who have undergone successful curative gastrinoma resection, PPIs may also be required because, in more than half of them, a hypersecretory state persists [180]. Interruption of PPI treatment (and consequent acid rebound) can have detrimental consequences, including severe peptic complications (strictures, perforations, etc.) [181]. Patient compliance to treatment is, therefore, crucial and should be regularly assessed. Over time, many patients could have the drug dose lowered [178].

The efficacy and safety of PPIs has revolutionized the management of ZES, so that total gastrectomy is no longer required. Long-term antisecretory treatment with PPIs has remained effective for more than 10 years, without development of tachyphylaxis or any dose-related adverse effect [182]. Even at the high doses required for patients with ZES, PPIs have a notable record of safety. An analysis of ZES patients can provide important insights into some of the safety issues, concerning long-term acid suppression [183].

### PPIs for stress ulcer prophylaxis (SUP)

#### Summary of the current evidence

*PPIs are the drugs of choice for acid suppression in SUP. The risk of bleeding in intensive care unit (ICU) is reduced by some 60 % in patients receiving SUP compared with those treated with placebo or no prophylaxis. Routine prophylaxis, however, is not justified by current evidence. SUP should be withheld in the majority of hospitalized patients, unless they have multiple risk factors, since only those at risk of clinically important bleeding (CIB) are most likely to benefit from preventive strategies. Educating clinicians to follow SUP guidelines can improve the cost-effectiveness of PPI use in this clinical setting.*

Stress-related mucosal disease (most commonly referred to as stress ulcer) is an acute condition that can be detected endoscopically in the majority (75–100 %) of critically ill patients, within 24 hours of admission to an ICU. However, the incidence of CIB from stress ulcer in the ICU population is low, the pooled figure from the most recent trials being some 1 % [184]. The incidence has improved substantially over recent decades, likely thanks to better overall ICU care. However, the mortality rate among patients with CIB was 48.5 %, which is significantly higher than that (9.1 %) of those without such bleeding [185], showing that stress-related mucosal disease can be a deadly condition.

Critically ill patients are at increased risk of developing stress-related mucosal disease and subsequent stress-ulcer bleeding as a result of both their underlying disease and therapeutic interventions. There are some well-established strong, independent risk factors for stress-related mucosal disease, respiratory failure and coagulopathy being the most relevant ones, with an OR of 15.6 and 4.3, respectively [184]. Other important factors include acute renal or hepatic failure, sepsis, hypotension, severe head or spinal cord injury, thermal injury involving more than 35 % of the body surface area, acute lung injury, major surgery (lasting more than 4 hours), and history of GI bleeding [186].

Although no single study or meta-analysis has reported a decrease in the overall mortality related to SUP, guidelines from the American Society of Health-System Pharmacists [187] and Surviving Sepsis Campaign [188] recommend routine prophylaxis with acid suppressive therapy for high-risk patients. The rationale for this recommendation relies on the finding that important GI bleeding is strongly associated with prolonged ICU stay and increased mortality [189]. Indeed, three large meta-analyses found that the risk of bleeding in ICU is reduced by some 60 % in patients receiving SUP compared with those treated with placebo or no prophylaxis [190–192]. Therefore, SUP has become the standard of care in the ICU, sometimes irrespective of the presence of risk factors. The benefit of SUP using real-world data is, however, not easy to estimate because of the lack of a control group.

H_2_RAs have also been found effective in preventing CIB [186] and are the preferred acid lowering drugs in some ICUs [193] despite the efficacy of PPIs being significantly better. Over recent years, all meta-analyses [194–197] except one [198] confirmed the superior efficacy of this class of drugs. No studies showed a statistically significant difference in the rate of severe complications, such as nosocomial pneumonia, since these associations were not reported outcomes in any of the RCTs assessed. A recent observational study [199] found that PPI treatment for SUP in critically ill patients is associated with a risk of *C. difficile* infection higher than that observed with H_2_RAs (6.7 % vs. 1.8 %).

SUP should be withheld in the majority of hospitalized patients, unless they have multiple risk factors [184, 186]. Indeed, as outlined by current practice guidelines [187, 188], any risk factor different from those mentioned above does not independently predispose a patient to stress ulcer bleeding. Moreover, a recent meta-analysis suggests that patients receiving enteral nutrition may not require SUP [200]. On the other hand, its implementation might be associated with an increased risk of infectious complications, such as nosocomial pneumonia and *C. difficile*-associated diarrhea. Patients with liver cirrhosis may actually have an increased mortality rate if treated with PPIs [201].

Nevertheless, several studies demonstrate that, in many non-ICU patients lacking an indication for SUP, acid suppressive therapy is started upon hospital admission [202]. A recent study [203], while confirming that PPI use for SUP has spread inappropriately to low-risk patients, found that more than 50 % of admitted PPI users were inadvertently prescribed a PPI at discharge, without a real medical need for acid suppression.

In summary, PPIs represent the drugs of choice for acid suppression in SUP. However, routine prophylaxis is not justified by current evidence. Only patients at risk of CIB are likely to benefit from preventive strategies. Educating clinicians to follow SUP guidelines can improve the cost effectiveness of PPI use in this clinical setting.

### PPIs for dyspepsia

#### Summary of the current evidence

*PPI therapy in both uninvestigated and functional dyspepsia (FD) is widespread. Indeed, these drugs represent a key component of all the currently employed* H. pylori *eradication regimens. The search for and eradication of the infection is the first line therapy in the young dyspeptic patient without alarm symptoms. In those patients with persisting symptoms despite successful eradication or naïve-uninfected patients with epigastric pain syndrome (EPS), short-term 4–8 week PPI treatment should be attempted. Finally, PPI co-therapy is indicated in patients with NSAID-associated dyspepsia, also with the aim of preventing GI events.*

Dyspepsia is a common GI condition seen in clinical practice. It is not a single disease, but rather a complex of symptoms referable to the upper GI tract that often overlaps with other disease entities. In front of a patient with dyspeptic complaints, physicians should carefully evaluate the history and perform a physical examination in order to assume that symptoms arise from the upper GI tract [204]. If the patient is young (< 45 years), and there are no alarm symptoms, endoscopy and/or functional investigations are usually not performed, the condition is labeled as “uninvestigated” dyspepsia and treatment is empiric. On the contrary, if endoscopy does reveal a structural abnormality, management of dyspepsia relies on treatment of the underlying disease (e.g., PU, reflux esophagitis, or malignancy). When endoscopy is negative (which is the case in more than 70 % of patients with dyspeptic symptoms), FD could be considered and, provided Rome IV criteria are fulfilled [205], the final diagnosis can be confirmed [204].

FD is characterized by a continuous or frequently recurring epigastric pain or discomfort centered in the upper abdomen for which no organic cause can be determined. According to the most updated Rome IV classification, dyspepsia is subdivided into postprandial distress syndrome (PDS, including fullness, early satiety, nausea) and epigastric pain syndrome (EPS, including epigastric pain or epigastric burning) [205]. However, symptom overlap with either GERD or irritable bowel syndrome is not infrequent [206, 207].

FD is widely prevalent in the general population − up to 15 % in Italy [208] − so that FD patients are frequently managed in clinical practice by both general practitioners and gastroenterologists. Since the etiology of FD remains unclear and is probably heterogeneous, no definite single treatment is currently available for these patients [204]. Indeed, different therapeutic approaches have been proposed.

Since some drug classes (e.g., NSAIDs, calcium channel blockers, corticosteroids, ACE inhibitors, and methylxanthines) can induce dyspeptic symptoms [209], a careful evaluation of the current drug therapy is of paramount importance: dyspeptogenic medications should be withdrawn whenever possible. In patients with NSAID-associated dyspepsia, PPIs are effective and should be given also with the aim of preventing adverse GI events (*see below*).

The role of *H. pylori* in FD is supported by data from meta-analyses showing that *H. pylori* eradication resulted in a statistically significant benefit compared with placebo (relative risk of remaining dyspeptic 0.90; NNT = 13) [210, 211]. However, short-term benefit is often not evident since symptom relief becomes significant only some (up to 6) months after successful cure of the infection [212]. Furthermore, PPIs represent a key component of all the commonly used eradication regimens (triple, quadruple, sequential, concomitant or hybrid therapies).

It has been estimated that the *H. pylori* “test and treat” strategy is cost-effective in those regions where prevalence of the infection is > 20 % [213], as in Italy, and that the advantage persists at long-term follow-up [214, 215]. Unfortunately, no predictive factors for clinical benefit have been identified, so that eradication treatments should be attempted in all dyspeptic patients. *H. pylori* should be investigated with either non-invasive tests or upper endoscopy, according to the age of the patient and the presence of alarm symptoms [148].

In patients with dyspeptic symptoms persisting despite successful eradication or naïve uninfected patients with EPS, PPI therapy can be attempted, an approach achieving a success rate of 34 % (NNT = 10, 95 % confidence interval, 7–33) [216]. PPIs are particularly effective when overlapping reflux symptoms are present, while no significant benefit occurs in dyspeptic patients with PDS [217]. It is worth emphasizing that the effect of PPIs in FD occurs at standard doses and, since meta-analyses found no dose-response effect [216, 217], escalating the dose in non-responders to standard doses should not be considered. If breakthrough symptoms occur, antacids or alginate-containing formulations may be used [216]. Differentely from GERD, a long-term therapy with PPIs in FD is not indicated [217]. After successful treatment, a tapering strategy rather than abrupt discontinuation is preferred [217]. Although symptoms may recur in nearly 70 % of patients within 1-year follow-up [82], re-starting treatment only in these patients is more advantageous than a continuous and expensive treatment, prescribed in all cases.

Dyspeptic symptoms are common in GERD patients, especially those with frequent reflux related symptoms. In these patients, epigastric pain, belching, bloating, and early satiety were found to improve on PPI therapy, conversely from nausea and vomiting, which did not benefit from acid suppression [218].

In addition to suppressing acid secretion, PPIs can also inhibit gastric motility and delay emptying rate [219] and, as a consequence, dyspeptic symptoms may actually be worsened by PPI therapy or, alternatively, new symptoms (especially postprandial fullness) may arise during treatment. If this is the case, patients could be switched to the H_2_RAs, ranitidine or nizatidine, which, in addition to their antisecretory activity, display a cholinergic-like activity [219] and have been shown to accelerate gastric emptying [220]. On the other hand, a Cochrane meta-analysis [216] showed that H_2_RAs are better than placebo in achieving symptom relief in patients with FD.

### PPIs for NSAID-associated symptoms and lesions

#### Summary of the current evidence

*Standard dose PPIs are indicated for patients taking non-selective NSAIDs at risk for upper GI complications (bleeding and perforation) and for those given selective cyclooxygenase (COX-2) inhibitors having had an episode of previous GI bleeding. In both non-selective and COX-2 selective NSAID users, PPI therapy reduces upper GI symptoms, in particular dyspepsia. However, NSAID-induced adverse events in the lower GI tract are not prevented by PPIs.*

NSAIDs are amongst the most widely used classes of drugs. Although they are very effective medications, their use is associated with a broad spectrum of adverse effects in the liver, kidney, cardiovascular (CV) system, skin, and gut [221]. GI adverse effects are the most common and include a wide clinical spectrum ranging from dyspepsia, heartburn, and abdominal discomfort to more serious events such as PU with life-threatening complications, including bleeding and perforation [222].

Since symptoms are not a reliable indicator of mucosal damage, it is important to identify factors that predict the risk of GI events in NSAID users. The risk factors for upper GI bleeding (UGIB) associated with NSAID use have been well defined by several studies [222]. Among them, the most important are prior history of complicated ulcer and age. Older age is common in NSAID users and those aged above 65 years carry a risk similar to those with a history of PU. Advancing age increases the risk by about 4 % per year, probably because of the presence of other associated risk factors [223]. The presence of multiple risk factors greatly increases the risk of GI complications [222]. The role of *H. pylori* infection in patients taking NSAIDs and the potential benefit of eradication on upper GI risk in infected NSAID users has been controversial. However, eradication of associated *H. pylori* infection is beneficial when starting treatment with NSAIDs or aspirin, especially in the presence of an ulcer history [90, 224].

An often forgotten risk factor for upper GI complications is represented by drug combinations with NSAIDs [225]. While the role of steroids, antiplatelet drugs, and anticoagulants is long known, the synergistic effect of selective serotonin reuptake inhibitors (SSRIs) has until recently been overlooked. Over the past 15 years, several epidemiologic studies, summarized by three recent meta-analyses [226–228], have shown an association between SSRI use and the occurrence of UGIB, and found that this risk is further increased among patients, who concomitantly use NSAIDs [229, 230] and/or hold *H. pylori* infection [231], while it is lowered by concomitant PPI intake [227, 232]. The most plausible mechanisms underlying this detrimental effect include a marked decrease in serotonin platelet content, with consequent impairment of platelet aggregation in response to injury and prolongation of bleeding time as well as an increase in gastric acid secretion, with potential ulcerogenic activity [233, 234]. When given with NSAIDs, SSRIs may inhibit their metabolism, raising their blood levels and – through impairment of the hemostasis – may promote more severe bleeding. Since the concomitant use of both these drugs results in a significantly higher risk of UGIB than either drug alone [229, 230], this combination should be avoided whenever possible and, if unavoidable, adequate gastroprotection should be adopted from the very beginning [235].

GI symptoms usually develop within the first few days of starting a NSAID therapy and can actually occur with the first dose of the drug. Although some studies have suggested that the first 2 months of treatment represent the period of greatest risk for complications with a relative risk of 4.5 %, available evidence (from both RCTs and observational studies) shows that the risk of GI complications is constant over time, either during short-term or long-term NSAID use [170]. Therefore, even a short course of NSAID therapy (e.g., for postoperative pain or acute musculoskeletal injury) carries a risk of GI complications similar to that of long-term treatment. As a consequence, prevention strategies should be implemented regardless of the duration of therapy, especially in patients with more than one risk factor (i.e., at high GI risk).

All RCTs have shown that PPIs are more effective than H_2_RAs in both preventing and treating gastroduodenal lesions [236]. The reasons underlying the superiority of this class of antisecretory drugs have been clarified by preclinical and clinical pharmacological studies indicating that degree and duration of acid inhibition are both important factors in determining their efficacy in the prevention of NSAID injury [237]. They also reduce upper GI symptoms associated with both COX-2 selective and non-selective NSAID use [236]. Due to the long half-life and entero-hepatic circulation of several NSAIDs, a split dose PPI might be useful; there is, however, no evidence for the clinical usefulness of this regimen.

COX-2 selective NSAIDs (often incorrectly referred to as coxibs^1^) have an improved upper GI safety profile compared to traditional (non-selective) compounds, as extensively shown in endoscopy and clinical outcome studies [238–240]. The evidence is strong, with consistent reductions in events of about 50 % in large RCTs, meta-analyses of RCTs, and large observational studies in clinical practice [238]. Among patients with a prior ulcer bleed, treatment with a COX-2 inhibitor or an NSAID plus PPI is still associated with a clinically important risk of recurrent ulcer bleed (some 10 %) [222]. In these patients, the combination of a PPI and a COX-2 inhibitor reduces the risk of upper GI bleeding compared to that of COX-2 inhibitor alone [28, 236]. A very recent network meta-analysis indeed found that this drug combination represents the best strategy to prevent ulcer complications [241].

In addition to the upper GI tract, the NSAID-induced damage extends beyond the duodenum. Since NSAID-induced intestinal injury involves non-acid-related mechanisms [222], co-administration of PPIs does not prevent NSAID-induced intestinal damage but might actually aggravate it [28, 170, 242, 243], most likely by inducing dysbiosis [244]. Furthermore, NSAID-associated lower GI bleeding is not prevented by PPI co-administration [245].

While the better upper GI safety of COX-2 selective agents over traditional NSAIDs is well established, their individual lower GI tolerability is less well evidenced and appears to differ. Both endoscopic and video-capsule studies have shown that celecoxib displays a better intestinal tolerability compared to an NSAID plus a PPI [28]. The good upper and lower GI safety profile was confirmed by the large CONDOR and GI-REASONS trials [28]. However, this benefit is partially lost when this COX-2 selective inhibitor is combined with a PPI [243].

On the other hand, over recent years, great attention has been focused on CV adverse effects of COX-2 selective inhibitors, which prompted a re-evaluation of the CV (and global) safety profile of traditional NSAIDs. Current evidence suggests that non-selective and COX-2 selective inhibitors display a similar incidence of these adverse effects, but with molecule-specific quantitative differences between the various drugs [246, 247].

NSAIDs are an essential part of the therapeutic armamentarium despite their well characterized GI and CV risk profiles. Physicians should not prescribe NSAIDs before taking a careful history and doing a physical examination so that they can acquire the information they need to balance risks and benefits for individual patients. When GI and/or CV risk factors are present, appropriate preventive strategies (i.e., COX-2 selective inhibitors and/or PPI use as well low-dose aspirin) should be implemented from the very beginning and compliance to treatment assessed regularly, especially in the elderly [222]. Finally, the appropriateness of an NSAID prescription should be emphasized, i.e., to control inflammation and pain, rather than to control pain alone [248]; only then can we hope to limit the expanding NSAID epidemic.

### PPIs for corticosteroid users

#### Summary of the current evidence

*Corticosteroid therapy does not cause damage to the gastroduodenal mucosa, but can enhance the GI risk associated with NSAID use. Therefore, unless patients taking corticosteroid therapy have a PU or are under concomitant NSAID therapy, mucosal protection with a PPI is not routinely indicated.*

Contrary to NSAIDs, corticosteroids do not cause any direct injury to the gastroduodenal mucosa [249], and indeed some experimental evidence actually suggests a mucosal protective effect [250, 251]. These drugs may, however, increase the GI risk of NSAID therapy and may hamper the healing of idiopathic or iatrogenic ulcers [252]. The association between corticosteroid use and GI adverse events in patients with risk factors other than NSAID use remains controversial. Indeed, some studies reported an increased risk of PU complications in corticosteroid users, while other investigators failed to demonstrate such an association after adjustment for confounding factors [253–257]. A meta-analysis also failed to show any significant risk for gastric or duodenal ulcers in patients receiving corticosteroid treatment compared to controls [258]. It is worthwhile emphasizing that the design of the studies included in the meta-analysis was quite heterogeneous as was the type of patients selected (outpatients or inpatients, presence of comorbidity and co-therapy) as well as PU definition. However, a systematic review of available meta-analyses as well as of published case–control studies reached the same conclusion [259].

A more recent systematic review and meta-analysis of 159 studies, appeared between 1983 and 2013, on GI bleeding and perforation in corticosteroid users [260], found that corticosteroid therapy may increase the risk of GI events (OR = 1.43) *only* in hospitalized patients. Here again, the diversity of GI bleeding definitions (widely varying from occult blood in stool to bleeding requiring transfusion or hospital stay) as well as the heterogeneity of the patients included do not allow drawing clinically relevant conclusions [260]. Taking these considerations into account, no evidence currently supports PPI therapy as prophylaxis for corticosteroid use in the absence of concomitant NSAID therapy.

In summary, PPI co-therapy is not routinely indicated in patients taking corticosteroids unless they have a history of PU or are taking NSAIDs. In hospitalized patients on corticosteroid therapy, prophylaxis against stress ulcers could be limited to those with a history of PU, clotting impairment or requiring mechanical ventilation for more than 48 hours [261]. Despite corticosteroids increase the risk of GI bleeding in patients with either diverticular disease of the colon or acute ischemic stroke [262, 263], PPI therapy is not expected to exert any preventive effect on eventual drug-induced GI bleeding in these patients.

### PPIs in patients taking anti-platelet or anti-coagulant therapy

#### Summary of the current evidence

*Standard dose PPI therapy is advised for gastroprotection in all patients on anti-platelet therapy who are at increased risk of gastrointestinal bleeding (age > 65 years or concomitant use of corticosteroids or anticoagulants or history of PU). International Normalized Ratio monitoring is required when starting or stopping PPI therapy in vitamin K antagonist users. In patients receiving clopidogrel or vitamin K antagonists, choosing PPI lacking interference with the hepatic CYP450 enzymes might be preferred. No demonstrated interaction exists between PPIs and the novel oral anticoagulants.*

Based on a documented efficacy, anti-platelet therapy (aspirin < 300 mg/daily, ticlopidine 100 mg/daily, clopidogrel 75 mg/daily) is widely used for both primary and secondary prevention of CV and cerebrovascular ischemic events [264, 265]. However, anti-platelet drugs may cause adverse GI events (gastroduodenal ulcerations/erosions, overt bleeding, occult bleeding, and – only seldom – a perforation), with a definite probability of death, particularly in the elderly [266]. Therefore, gastroprotection is advised in those patients at increased GI risk during anti-platelet therapy. Increased risk factors include age above 65 years, concurrent use of steroid/anticoagulant therapy, or history of PU [222, 255, 267–269]. Presence of relevant co-morbidities (heart failure, renal impairment, stroke, diabetes, on-going malignancy) and smoking are additional risk factors for both GI events and related mortality [255, 268–270]. Standard PPI-dose is the most effective gastroprotective therapy [271, 272]. Regrettably, PPI therapy is not effective in preventing bleeding lesions in either small intestine or colon induced by anti-platelet drugs, for which no protective strategies are today available [269, 273, 274].

A matter for concern is the interaction between PPIs and clopidogrel. Based on pharmacokinetic studies, PPI therapy reduces the efficacy of clopidogrel by interfering with the hepatic CYP2C19-based activation [275]. However, the clinical relevance of such a phenomenon is largely controversial. Indeed, a panel of experts of the European Society of Cardiology recently suggested that there is no conclusive evidence to discourage PPI use with clopidogrel due to a potential increased risk of ischemic events [276]. Further, the only prospective study (the COGENT trial) demonstrated that omeprazole significantly reduces the rate of composite GI events but did not show any increase in the composite CV events in patients at high CV risk [277]. Additionally, a recent meta-analysis [278], while confirming that the pharmacodynamic interaction between PPI and clopidogrel has no clinical significance, actually suggests that PPIs are a marker of increased CV risk in patients taking clopidogrel rather than a direct cause of worse outcomes. Nevertheless, PPIs that lack inhibition of CYP2C19 (i.e., pantoprazole or rabeprazole) might be preferred in clopidogrel users [276]. No significant interaction between PPIs and the new antiplatelet agents (prasugrel or ticagrelor) has been documented [276].

Co-administration of aspirin and clopidogrel is associated with synergistic effects in causing serious GI bleeding. Indeed, epidemiological studies have invariably shown that combination of two antiplatelet drugs produces significant excess risk of UGIB [225, 279]. PPI co-treatment is also effective in reducing the risk of UGIB in patients receiving dual antiplatelet therapy [280, 281]. It is worthwhile mentioning that – among patients with dual antiplatelet therapy and PPI co-therapy – GI bleeding episodes are more frequent in the lower GI tract [282]; this changing pattern of bleeding likely reflects the success of gastroprotection [283].

Anticoagulants, either vitamin K antagonists or novel oral anticoagulants, including dabigatran, rivaroxaban and apixaban, do not cause gastroduodenal mucosa injury *per se*. These medications may, however, facilitate bleeding of pre-existing PUs. While gastroprotection is generally not advised, unless a concomitant anti-platelet or NSAID therapy is prescribed, a very recent retrospective cohort study found that PPI co-therapy is associated with reduced risk of warfarin-related upper GI bleeding. As expected, the risk reduction was greatest in patients also taking antiplatelet drugs and/or NSAIDs, but was still significant in those without concurrent use of these medications [284].

In patients under acid suppression because of gastroprotection or any acid-related disease, intensified International Normalized Ratio monitoring is recommended since PPIs may potentiate vitamin K antagonist-induced anticoagulation, most likely due to facilitated gastric absorption of warfarin [276]. When acenocoumarol is used as an anticoagulant, some caution is needed when prescribing PPIs (in particular omeprazole, esomeprazole, and lansoprazole) because of potential DDIs [285]. No clinically significant interaction during PPI and novel oral anticoagulant co-administration occurs, so that dabigatran-related dyspepsia may be safely treated with PPIs [276].

### PPIs for PU bleeding

#### Summary of the current evidence

*Endoscopy is the mainstay of treatment of PU bleeding. However, PPI therapy – after endoscopic hemostasis – reduces the risk of re-bleeding, the requirement for surgery, and mortality in high-risk patients. Pre-endoscopic administration of a PPI can be useful in downgrading stigmata of recent hemorrhage, thereby reducing the need for endoscopic hemostatic procedures.*

The goal of medical therapy for bleeding ulcers has been traditionally aimed to maintain a sustained intragastric pH (> 6 units), in order to promote platelet aggregation as well as clot formation and stability [286, 287]. Indeed, platelet function is impaired at low pH [288], and pepsin promotes clot lysis below pH 5 [289].

 Although endoscopic therapy is able to achieve hemostasis in most patients, recurrent bleeding is not uncommon [290, 291]. Several meta-analyses [292–294] have shown that adjuvant treatment with a PPI after endoscopic hemostatic therapy reduces the risk of re-bleeding and the requirement for surgery after ulcer bleeding but has no benefit on overall mortality, an effect seen only in Asian trials and in patients with active bleeding or a non-bleeding visible vessel. In addition, treatment with a PPI produces small, but potentially important, reductions in transfusion requirement and length of hospitalization [295, 296]. Although current guidelines [297–299] recommend a regimen of an intravenous (i.v.) bolus followed by a continuous infusion of PPIs, a recent meta-analysis [300] found that the efficacy of continuous and intermittent PPI therapies were comparable. Whether low-dose or oral PPIs can substitute high-dose PPIs after endoscopic hemostasis is controversial. In a recent meta-analysis from Taiwan [301], investigators concluded that low- and high-dose regimens were equivalent. However, they included trials with a small number of patients and patients with ulcers showing low-risk stigmata or even clean-base ulcers. Similarly, some trials [302, 303] reported that the efficacy of oral PPIs is comparable to that of intravenous PPIs, but the results were combined from open-labeled trials with limited sample size. Furthermore, different oral regimens were combined together and compared with different intravenous regimens. It is also worth mentioning that most studies included in this setting have been performed in Asian patients [304].

Currently available PPIs are not able to maintain the intragastric pH above 6 for prolonged periods [305]. As a consequence, intravenous infusion has often been used in clinical studies. Intravenous esomeprazole is faster and more effective in raising intragastric pH than i.v. lansoprazole [306] or i.v. pantoprazole [307–309]. Even by the oral route, esomeprazole 40 mg, which provides the most effective control of intragastric pH amongst the class [54, 55], achieves greater acid inhibition than intravenous pantoprazole (40 mg/daily) on both day 1 and day 5 [310]. The Peptic Ulcer Bleed study, involving 91 hospital emergency departments in 16 countries, showed that high-dose intravenous esomeprazole (80 mg, followed by 8 mg/h infusion, over 72 hours), given after successful endoscopic therapy to patients with high-risk stigmata of PU bleeding, reduced recurrent bleeding at 72 hours and had sustained clinical benefits for up to 30 days while patients were on maintenance oral esomeprazole (40 mg daily) [311].

PPI treatment, initiated prior to endoscopy in patients with upper GI bleeding, significantly reduces the proportion of patients with stigmata of recent hemorrhage at index endoscopy: pooled rates were 37.2 % and 46.5 %, respectively (OR = 0.67) [312, 313]. Although there is no evidence that PPI treatment affects clinically important outcomes (mortality, re-bleeding, or need for surgery), pre-endoscopic (or even pre-hospital), oral or intravenous, administration of a PPI could downgrade high-risk stigmata of recent hemorrhage. This might increase the success of endoscopic hemostatic therapy and/or reduce the requirement for it.

### PPIs in patients with cancer

#### Summary of the current evidence

*In cancer patients, PPI use could be indicated to treat and/or prevent chemotherapy-induced GERD and gastroduodenal ulceration, with accompanying symptoms. Patients with GI mucositis or dysphagia might also benefit from these drugs. Due to the low number or poor quality of the available studies, the evidence supporting these indications is low.*

Amongst the adverse effects of cancer chemotherapy, GI symptoms are the most common and have the greatest impact on the quality of life [314–316]. Fewer than 20 % of affected patients are referred to a GI specialist [317] since clear management algorithms and routine referral pathways are not in place due to limited research in this topic. As a consequence, some treatable symptom complexes go unrecognized and/or ineffective and potentially harmful treatments are prescribed. Sometimes, persistent symptoms do compromise or prevent ongoing anticancer treatment.

While several guidelines for management of the GI adverse effects of cancer chemotherapy do exist [318–324], only the European Society for Medical Oncology [318] and Multinational Association of Supportive Care in Cancer/International Society of Oral Oncology [324] clinical practice recommendations address gastroesophageal mucositis. Different types of cancer therapy alter the integrity of the GI mucosa [325] as well as the microbial flora that inhabit the oral cavity and the gut [326]. These treatments also affect the amount and composition of saliva [327]. In addition, they negatively impact on epithelial turnover and maturation, leading to impairment of the mucosal barrier [325]. Up to 40 % of patients undergoing treatment with gemcitabine or S-1 (an oral pro-drug of 5-fluorouracil) for pancreatic cancer complained of GERD-related symptoms [328], and endoscopy revealed the presence of severe mucosal lesions (multiple gastric erosions, diffuse erosive gastritis, gastric or duodenal ulcer) in 46 % of patients after a single cycle of chemotherapy (with cisplatin plus etoposide) [329]. Gastroduodenal ulcers are extremely frequent (up to 100 %) in patients submitted to hepatic intra-arterial chemotherapy [330, 331]. Even the more recent molecular-targeted agents are not devoid of GI adverse events [332]. For instance, ulceration and perforation of the stomach and bowel are well-known complications of bevacizumab (a humanized monoclonal antibody against vascular endothelial growth factor) [333, 334].

The rationale for acid suppression in patients undergoing cancer chemotherapy stems from the evidence that cytotoxic treatment damages the gastroesophageal mucosa, but does not affect the acid producing capacity of parietal cells. Therefore, mucosal damage may result – at least in part – from the effect of an aggressive acid-peptic secretion on an already damaged mucosa [335]. British guidelines [320] suggest PPI use – amongst other therapeutic options – in the management of dysphagia and/or retching induced by cancer chemotherapy, but do not provide any indication about the dose and treatment duration. Acid suppression appears to be beneficial in the treatment of GI mucositis induced by chemotherapy [336] and radiation [337], while once daily PPIs seem to improve chemotherapy-induced GERD symptoms [328], but data are conflicting. Two large clinical trials [338, 339] have shown PPIs to be effective in the prevention of chemotherapy (cyclophosphamide methotrexate fluorouracil or 5-fluorouracil)-induced gastroduodenal injury and in reducing the incidence of heartburn and epigastric pain. In the more recent study [339], omeprazole was also highly effective in preventing delays in cancer treatment. No postponement of chemotherapy was required for patients treated with PPIs, while chemotherapy was delayed in some patients receiving placebo or an H_2_RA.

Several other indications for PPI use in cancer patients have been suggested and/or proposed [340]; they include postoperative symptom relief in patients after gastric or esophageal resective surgery, prophylactic use in patients treated with NSAIDs as analgesics in cancer pain, acute management of upper GI bleeding in patients with proximal GI cancer, prophylactic use in patients treated with palliative upper GI endoscopic stenting, and prophylactic use in cancer patients who are at high risk for PU disease. However, most of the above indications have not been formally tested in specific, well-designed RCTs. In addition, even for those evaluated in clinical studies (i.e., chemotherapy-induced GERD symptoms or gastroduodenal ulceration), the evidence is low and often derives from single studies or single centers. Nevertheless, up to 73 % of patients admitted to oncology or hematology units are treated with acid suppressants (PPIs or H_2_RAs) [341, 342], mostly for SUP or *unspecified* gastroprotection.

The Globocan survey [343] indicates that, in 2012, approximately 32.6 million people were living with cancer (within 5 years of diagnosis). It is therefore astonishing and disappointing that thousands of studies, often dealing with trivial GI symptoms and spending billions of euros, have been performed while very few have evaluated the potential indications of PPIs in cancer to provide physicians with the best way to cope with the GI adverse effects of chemotherapy or radiotherapy and relieve the suffering of these difficult patients.

Besides the several potential and alleged indications of PPIs in cancer patients, increasing evidence suggests, for this class of drugs, an anti-tumor effect (through the selective induction of apoptosis as well as an anti-inflammatory effect) [344]. They also exert a protection of cancer cells from developing chemo- or radiotherapeutic resistance [345]. Acidification of the extracellular compartment represents a conceivable mechanism of drug resistance in malignant cells. In addition, it drives proliferation and promotes invasion and metastasis [345]. Experimental evidence has shown that PPIs counteract tumor acidification (via inhibition of vacuolar H^+^ ATPase) and restore sensitivity to anticancer drugs. Moreover, early clinical data have supported their role as add-on medications to anticancer treatments in patients with osteosarcoma or breast cancer [346]. A recent large epidemiological study (the SPORE program [347]) found that patients with head and neck tumors, taking acid suppressants, had significantly longer overall survival compared to those who did not. Specifically designed clinical trials are ongoing to better characterize the role of PPIs as new therapeutic agents in cancer treatment. Therefore, at the current status of present knowledge, PPI use as adjunct to cancer chemotherapy should not be performed outside a clinical trial.

### PPI use in cirrhosis

#### Summary of the current evidence

*PPI use in patients with liver cirrhosis must be very cautious since there is no evidence of benefit except for downgrading esophageal ulcers after sclerotherapy or banding of esophageal varices. There is also some evidence that their use could be associated with development of spontaneous bacterial peritonitis*.

In clinical practice, PPI therapy is often used in cirrhotic patients, in the absence of acid-related disease, to prevent bleeding from hypertensive gastropathy [348]. This approach is not evidence-based and therefore not justified, also taking into account that acid secretion is markedly reduced in these patients [349].

A systematic review, performed in 2008, was unable to find any randomized clinical trials on acid-lowering drugs in the prevention of esophago-gastric variceal bleeding in cirrhotic patients. It is therefore not possible to establish whether these drugs are beneficial or harmful in this setting [350]. It is worth mentioning, however, that PPI use is associated with a microbiota shift and functional changes in the distal gut of patients with compensated cirrhosis, an effect that could drive or aggravate the pre-existing small intestine bacterial overgrowth [351, 352].

The only indication of PPIs, which is by the way weakly evidence-based, is the prevention and/or treatment of esophageal ulcers after sclerotherapy or variceal band ligation. The best available evidence supports the short-term use (maximum 10 days) of PPIs to reduce ulcer size, provided spontaneous ulcer healing is a concern. Practices such as high dose infusion and prolonged use should be discouraged until evidence of benefit becomes available [353].

When prescribing PPIs to cirrhotics, the benefit-to-harm ratio must be carefully evaluated. Indeed, PPI therapy seems to be a risk factor for the onset of spontaneous bacterial peritonitis. A meta-analysis [354] showed that, compared to cirrhotic patients not receiving PPIs, those taking these drugs had an increased risk (*N* = 3815; OR = 3.15; 95 % confidence interval, 2.09 − 4.74) of developing spontaneous bacterial peritonitis, an effect more evident in case–control studies than in cohort studies [355]. Another systematic review [356] also found an increased overall risk of bacterial infections (OR = 1.98). A more recent case–control study discovered that PPI use in cirrhotic patients increases the risk of development of hepatic encephalopathy in a dose-dependent fashion [357]. All these analyses were mainly based on retrospective studies. The only large, multicenter, prospective trial [358] with PPIs dealt with spontaneous bacterial peritonitis and found acid suppression not associated with an increased risk. Further studies are therefore needed to better clarify these clinically relevant issues.

### PPIs for pancreatic diseases

#### Summary of the current evidence

*PPIs do not affect the clinical course of acute pancreatitis (AP), such as the length of hospitalization and time to starting oral intake or pain relief and, as a consequence, they are not recommended routinely in this clinical setting. However, their use – as add-on medication to enzyme replacement therapy (ERT) – is indicated in patients with chronic pancreatitis (and other diseases characterized by exocrine pancreatic insufficiency), whose steatorrhea is refractory to ERT.*

AP is one of the most common GI disorders, requiring acute hospitalization worldwide, with a reported annual incidence of 13–45 cases per 100,000 persons [359]. AP is an inflammation of the pancreas; it is sometimes associated with a systemic inflammatory response that can impair the function of other organs or systems. The inflammation may subside spontaneously or may progress to necrosis of the pancreas and/or surrounding fatty tissue. The dysfunction may resolve or may progress to organ failure. As a consequence, there is a wide spectrum of disease from mild, where patients recover within a few days, to severe (10–15 %) with prolonged hospital stay, the need for critical care support, and a 15–20 % risk of death [360, 361]. In Italy, AP is more commonly a mild disease with a biliary etiology and a low (5 %) overall mortality rate [362]. Whatever the cause, it should be recognized that AP is a systemic disease and not simply a disease of the pancreas. Thus, this common, potentially deadly, condition requires up-to-date evidence-based treatment.

AP is characterized by pancreatic and peripancreatic fat injury in part mediated by autodigestive enzymes. Excessive stimulation of the exocrine pancreas was long thought to worsen AP [363] and this represented the rationale for using inhibitors of exocrine pancreatic secretion (e.g., somatostatin and its analogs) as potential therapies for AP [364]. Along the same lines, antisecretory agents might be able – via inhibition of acid secretion and secretin release, due to duodenal acidification [365] – to contribute to the inhibition of pancreatic secretion.

Despite omeprazole being unable to inhibit amylase release from isolated pancreatic acini [366], pantoprazole appears capable of reducing tissue infiltration of inflammatory cells and acinar cell necrosis in rats with experimentally-induced pancreatitis [367], an action likely mediated by a reduced expression of inflammatory and adhesive proteins, key mediators in the pathogenesis of AP. The only clinical trial to date [368] found that treatment with pantoprazole does not affect the clinical course of AP such as the length of hospitalization, time to starting oral intake or pain relief. In addition, two retrospective studies found that PPI use does not affect clinical outcomes of patients with severe AP [369] or prevent post- endoscopic retrograde cholangiopancreatography pancreatitis [370]. As a consequence, most international guidelines on the management of AP do not even mention PPIs. The only exception is the Italian guideline [371], which clearly states that the routine use of PPIs is not recommended in patients with acute AP. This guideline and a former Japanese one [372] suggest that these drugs might be considered on a case-to-case basis, provided that specific indications, such as NSAID use, PU disease or bleeding, occur.

Chronic pancreatitis (CP) is a progressive, irreversible, fibro-inflammatory disease of the pancreas, in which the pancreatic secretory parenchyma is damaged and replaced by fibrous tissue, resulting in morphologic changes of the ducts and parenchyma. This process eventually culminates in permanent impairment of exocrine and endocrine function of the pancreas. The clinical manifestations of CP include abdominal pain as well as steatorrhea, weight loss, and malnutrition, all resulting from the loss of adequate enzyme and bicarbonate secretion [373, 374], and the so-called pancreatogenic diabetes, arising from loss of β-cell function [375, 376].

Chronic disabling pain, which poses a major detriment to the quality of life, is present in nearly 80–90 % of patients with CP. Although two meta-analyses found no real benefit of ERT [377], the role of pancreatic enzymes in mitigating abdominal pain still remains unclear and some experts recommend a 6-week trial of non-enteric-coated pancreatic enzymes [378].

Together with pain, most patients with advanced CP will develop pancreatic insufficiency at some point, secondary to loss of pancreatic parenchyma. Pancreatic lipase secretion is lost faster than other secretions. It is well known that steatorrhea (due to decreased luminal hydrolysis of dietary fat) develops only when the lipase levels drop to less than 10 % [379]. However, this does not minimize the importance of amylase and trypsin, which should be an integral part of pancreatic enzyme supplementation. Traditionally, ERT is indicated in patients with steatorrhea (fecal fat greater than 15 g/day) and weight loss [380]. Supplemental pancreatic enzymes alleviate diarrhea and maldigestion, associated with pancreatic exocrine insufficiency and also aid in maintaining normal nutrition, thus improving the quality of life [380].

Pancreatic enzymes in tablet form are susceptible to inactivation by stomach acid, therefore limiting their activity in the duodenum. The secretion of bicarbonate, which maintains an alkaline pancreatic juice, is dramatically reduced in most patients [380]. In addition, during the post-prandial period, the pH of the stomach and the upper small intestine is significantly decreased [381]. When postprandial pH in the small intestine becomes 4 or lower, bile acids precipitate and digestive enzymes (lipase in particular) lose their activities. Strategies to circumvent this include administering higher amounts of pancreatic enzymes and increasing gastric pH with the use of a PPI.

The favorable effects of antisecretory drugs on abolishing steatorrhea are primarily caused by reducing acid and volume of secretion. The end results of these actions are to elevate the pH in the stomach and duodenum, and facilitate the delivery and concentration of lipase in the duodenum. Another important effect is, however, the reduction of duodenal volume flow, which in turn increases the concentration of intraduodenal lipolytic activity [382]. Enteric-coated formulations protect pancreatic enzymes from the low pH in the stomach, allowing enzymes to maintain their potency when they reach the duodenum. Enteric-coated enzymes are indeed released in the duodenum, where the pH exceeds 5.5 units. Despite these formulations being resistant to breakdown by gastric acid, some studies have shown improvements in fat absorption when they are administered with concurrent acid suppression therapy [380].

The use of PPIs is recommended by international guidelines for CP and/or pancreatic exocrine insufficiency [383–391]. The Italian guidelines [384] clearly state that addition of PPIs is recommended only in patients with refractory steatorrhea and that acid suppression is not indicated in patients with an adequate response to ERT. These recommendations encompass the main diseases associated with exocrine pancreatic insufficiency (like acute pancreatitis, chronic pancreatitis, pancreatic cancer, cystic fibrosis, celiac disease, irritable bowel syndrome, etc.), defined as an inadequate pancreatic enzyme activity to digest food, generally due to either insufficient enzyme production, insufficient enzyme activation, or to early enzyme degradation [386, 392].

### Safety concerns with long-term PPI therapy

#### Summary of the current evidence

*Being very effective and considered very safe, PPIs are often prescribed inappropriately, especially in the elderly. The tolerability of PPIs is generally good, with an adverse event rate of 1–3 %. Some adverse effects are plausible and predictable; others are idiosyncratic, unpredictable, and rare. Overall, the benefits of PPI treatment outweigh the potential risks in most patients, who have a relevant and appropriate indication*.

Although overuse and misuse may challenge the safety profile, the tolerability of PPIs has been remarkably good. Adverse events generally occur at a rate of 1–3 %, without any significant differences among PPIs. Untoward effects most commonly include headaches, nausea, abdominal pain, constipation, flatulence, diarrhea, rash, and dizziness. Long-term studies indicate a tolerability profile similar to that found in short-term trials [22–25].

PPI-related adverse events involve the GI tract as well as other organs and systems. The majority of these events have been summarized in comprehensive reviews, to which the reader is referred [393–399]. The potential risks of long-term PPI therapy, along with the respective evidence summary, are outlined in Tables 1 and 2.

**Table 1:** Concerns about long-term therapy with proton pump inhibitors (PPIs): Digestive System

Theoretical Concern	Evidence Summary
Consequences of long-term PPI-induced hypergastrinemia	• PPI-induced hypergastrinemia is enhanced in *H. pylori*-infected patients [448], where the antisecretory effect is increased • No controlled human data support the increased risk of gastric cancer [92, 449] • More and higher quality studies are needed to confirm or refute any causal link with gastric cancer [450, 451] • No data support the increased risk of colorectal cancer [452] • An increased frequency of fundic gland (inflammatory) polyps has been reported [451, 453] • Rebound acid hypersecretion (and increased frequency of acid-related symptoms) is of uncertain clinical relevance [83] • Despite unclear biological mechanism(s), two large observational studies [454, 455] reported conflicting results concerning the putative risk of pancreatic carcinoma in PPI users
Infectious consequences of long-term PPI-induced hypochlorhydria	• Growing evidence suggests that acid suppression increases the risk of enteric infections by *C. difficile* [456–458] and other pathogens [456, 459] • Increased *Candida* infections in the mouth, esophagus, stomach, and upper small intestine of PPI users have been documented [460] • PPI users are at increased risk of small intestinal bacterial overgrowth (SIBO) [461], while cirrhotic patients, taking these drugs, are at higher risk of spontaneous bacterial peritonitis [354, 355]
Non-infectious consequences of long-term PPI-induced hypochlorhydria	• According to a single case-control study [462], exposure to antisecretory drugs, including PPIs, was associated with an increased rate of subsequent diagnosis of celiac disease, but the biological plausibility is unclear
Dysbiosis	• Dysbiosis probably represents the most consistent adverse effect of PPIs, responsible – besides enteric infections and SIBO – for gas-related symptoms as well as aggravation of NSAID-enteropathy [463, 464]
Consequences of long-term PPI-induced hypochlorhydria on electrolyte and nutrient absorption	• No consistent effects on calcium or iron absorption have been reported [394, 395] • Severe symptomatic hyponatremia has been reported as a consequence of the syndrome of inappropriate ADH secretion [465] • Data support an increased risk of developing significant B_12_ deficiency [466], but this is a clinical concern only in elderly or malnourished patients
Idiosyncratic reactions to PPIs	• Magnesium intestinal transport is inhibited by PPIs and may lead to rare but potentially life-threatening hypo-magnesiemia [467, 468] • Lansoprazole-induced microscopic colitis has been described [469], with complete resolution after drug discontinuation; however, recent data suggest a class effect [470] • Despite some case reports, epidemiological studies showing an association between PPI intake and acute pancreatitis have given conflicting results [44]

**Table 2:** Concerns about long-term therapy with proton pump inhibitors (PPIs): Extra-digestive effects

Theoretical Concern	Evidence Summary
Infectious consequences of long-term PPI-induced hypochlorhydria	• More and higher quality studies are needed to confirm or refute any causal link with community-acquired pneumonia, especially in long-term users [471, 472]
Bone consequences of long-term PPI therapy	• PPI use is not associated with accelerated bone mineral density loss or osteoporosis [473], which are thought to be the underlying biological explanation for the modest increase in the risk of bone fracture [474–476]
Dementia and Alzheimer’s disease	• Although, in a mouse model, very high-dose PPI use increased the level of β-amyloid in the brain [477], human data linking these drugs to development of dementia are conflicting [478, 479] • The risk of development of Alzheimer’s disease in PPI users appears comparable to that of any incident dementia [480]
Delirium	• PPIs were found to be an independent factor associated with development of delirium in geriatric inpatients [481], likely reflecting poly-pharmacy and drug-to-drug interactions (DDIs)
Acute myocardial infarction (AMI)	• Since PPIs do not impair endothelial function [482], more and higher quality studies, which have to be free from confounders [483], are needed to confirm [484, 485] or refute [277, 278, 408] any causal link with AMI
Idiosyncratic reactions to PPIs	• PPIs appear to be the most common cause of drug-induced acute interstitial nephritis (AIN). After PPI withdrawal and corticosteroid therapy, almost all patients recovered a normal renal function [486, 487] • There is a small but definite increase in risk of chronic kidney disease in long-term PPI users, likely resulting from undiagnosed or residual PPI-induced AIN [487] • Polymyositis and other myopathies, including the life-threatening condition of rhabdomyolysis, have been described with all PPIs [488, 489] • Immediate and delayed hypersensitivity to PPIs, with cross reactivity amongst the members of the class, has been described [490]
PPI-drug interactions	• Acid suppression reduces absorption of levothyroxine, ketoconazole, itraconazole, atazanavir, cefpodoxime, enoxacin, and dipyridamole while increasing that of nifedipine, digoxin, and alendronate [400] • Concomitant use of some PPIs with clopidogrel attenuates the antiplatelet effect of clopidogrel, but may not be clinically relevant since there are no clinical differences in the risk for major adverse CV events [401–403] • Only a few drug interactions (e.g., with diazepam, warfarin, phenytoin, and methotrexate) involving PPIs (mainly omeprazole and lansoprazole) are of clinical significance [401, 403] • DDIs may be more frequent in some patient populations (e.g., AIDS or cancer) [403] • The degree of DDIs associated with PPIs and the respective clinical outcomes depend on different factors such as genotype status of CYP enzymes, ethnicity, and drug regimen [403–405]

Gastric pH is relevant for the absorption of several drugs and its modification by antisecretory therapy may significantly modify their pharmacokinetics [400]. PPIs also influence drug absorption and metabolism by interacting with adenosine triphosphate-dependent P-glycoprotein (e.g., inhibiting digoxin efflux) or with the CYP P450 enzyme system (e.g., decreasing simvastatin metabolism), thereby affecting both intestinal first-pass metabolism and hepatic clearance. A number of studies have shown that omeprazole (and, to a lesser extent, lansoprazole) carries a considerable potential for DDIs, since it has a high affinity for CYP2C19 and a somewhat lower affinity for CYP3A4. In contrast, pantoprazole and rabeprazole display a lower potential for DDIs [401, 402]. DDIs therefore represent a molecule-related effect rather than a class-effect [403].

These interactions are clinically relevant mostly for drugs with a narrow therapeutic index (e.g., diazepam, warfarin, antipsychotics, etc.) [401, 404]. In addition, PPI metabolism is very rapid in most Caucasian subjects (extensive metabolizers), so that their half-life ranges from only 0.5 to 2.1 hours [404]. Indeed, the prevalence of poor metabolizers, potentially at increased risk of drug interactions, is as low as 1.2–3.8 % in Europe as compared to 23 % in Asia [405]. This could explain why only few of the reported DDIs involving PPIs have been shown to be of clinical significance.

Recent studies have raised concerns about a possible adverse interaction between clopidogrel and PPIs (currently prescribed to patients who are receiving dual antiplatelet therapy to prevent upper GI bleeding) that could reduce the antithrombotic effect of the former and, therefore, lessen protection against CV events in high-risk patients. However, current evidence shows that, while concomitant use of some PPIs with clopidogrel does attenuate the antiplatelet effect of clopidogrel, this effect is unlikely to be clinically relevant [276, 278, 406, 407]. Conversely, denying PPIs to patients at GI risk would result in increased life-threatening GI bleeding [277, 408, 409].

PPIs are among the most widely used prescription drugs. Although alarms have been raised about their long-term safety, the preponderance of the evidence does not strongly support the concerns, publicized over the last few years and the benefit to harm ratio remains favorable.

The best available information on long-term safety of PPIs derives from the SOPRAN [74] and LOTUS [73] trials, comparing ARS with omeprazole or esomeprazole, respectively. Safety data were collected from patients during the 12-year period of the SOPRAN study (*n* = 298) and the 5-year period of the LOTUS study (*n* = 514). Serious adverse events and changes in laboratory parameters were analyzed. Across both studies, serious adverse events were reported at a similar frequency in the PPI and ARS treatment groups. Laboratory results, including routine hematology and tests for liver enzymes, electrolytes, vitamin D, vitamin B_12_, folate, and homocysteine, showed no clinically relevant changes over time. The only expected difference concerned gastrin and chromogranin A levels, which were elevated in the PPI group, with the greatest increases observed in the first year [410]. Despite a continued proliferative drive on enterochromaffin-like cells during esomeprazole treatment, no dysplastic or neoplastic lesions were found [411].

Based on the quality of the overall evidence, the benefits of PPI treatment outweigh the potential risks in most patients, especially if PPI use is based on a relevant and appropriate indication [6, 44]. On the contrary, patients treated without an appropriate therapeutic indication are only exposed to potential risks. Because PPIs are overprescribed in many patients, in particular for continued long-term use, the clinical effects should always be reviewed and justified attempts should be made to stop any therapy that may not be needed [397].

## Discussion

PPIs remain the leading evidence-based therapy for acid-related diseases, including GERD, PU disease, dyspepsia, NSAID-induced ulcer, *H. pylori* infection, and hypersecretory disorders such as ZES [1, 3, 4, 412, 413].

The strong evidence supporting PPI efficacy and a favorable safety profile has led to overuse of these drugs in many treatment arenas [11]. Surprisingly, despite more than 25 years of extensive literature addressing PPI therapy in upper GI disorders, inappropriate use remains consistently high both in hospital and in primary care [7]. In a recent US study [414], only 39 % of inpatients’ prescriptions were compliant to guidelines, with a difference between academic and non-academic hospitals (compliance being 50 % vs. 29 %, respectively). Prophylaxis of upper GI bleeding in low risk patients was the most common indication for non-compliant prescriptions, while that of guideline compliant prescriptions was treatment of dyspepsia [414].

The questionable and inappropriate PPI use in the absence of documented evidence, supporting clear indications, is likely due to the perception that many physicians have about PPI safety, which makes them forget to assess the harms and benefits of (especially long-term) therapy [415]. Several studies (for a review see [11]) have shown that physicians frequently do not review and document PPI indications, which often results in their long-term or even indefinite continuation.

There are two main concerns pertaining to PPI overuse and misuse: drug expenditure, which has risen dramatically in recent years, even after the introduction of cheaper generic formulations [416], and growing safety concerns [393–399, 417]. Despite their concentration within the secretory canaliculi of the parietal cell and their pharmacologic selectivity [418], PPIs also have a “dark side” [419]. Sir William Osler once famously commented that no drug has a single effect and these secondary actions range from mildly inconvenient to frankly dangerous [420] – PPIs are no exception.

Non-judicious PPI use is a matter of great concern in the elderly, who often have multiple comorbidities, are taking multiple medications, and are thus at increased risk of long-term PPI-related adverse outcomes. PPI-related adverse events involve the GI tract as well as other organs and systems. The potential risks of long-term PPI therapy, along with the respective evidence summary, have been summarized in the Safety section. While some concerns about the possible adverse effects of PPIs (including an increased risk of gastric carcinoids, gastric carcinoma, decreased absorption of minerals and vitamins) have been raised since their introduction in the late 1980s, more recently, the number of publications dealing with PPI-related adverse outcomes has steadily increased [393–398, 417]. It is worth mentioning, however, that the majority of studies are observational in nature and do not allow for establishing causality, but merely associations [421]. Residual bias is always a concern in observational studies, even after statistical adjustment, because all confounding factors cannot be recorded or even known. When effect sizes are small (relative risk/OR/hazard ratio < 2), it is not possible to determine whether the association is valid or represents the result of residual bias [422]. Hazard ratios for PPI use and some reported adverse effects (e.g., dementia, chronic kidney disease, any fracture, or community-acquired pneumonia) were all ≤ 1.5. Nevertheless, if a true causality exists, even small effect sizes can result in a meaningful risk for common interventions and conditions.

Despite PPIs carrying – like any other classes of drugs – some risks, they should not be denied to patients who are likely to benefit from them merely because of concerns about putative adverse effects. There is generally some equivalence between the acceptable burden of adverse effects and the severity of the illness being treated [420]. However, patients with acid-related diseases are often otherwise “healthy” subjects, who take drugs for a given condition. What level of undesirable effects would be acceptable for them and who will bear the cost of treating any ensuing iatrogenic disease? A number of simple and potentially effective preventive measures should be recommended for some (if not all) safety concerns in order to minimize them.

First of all, PPI therapy should be evidence based. Decisions on whether or not to initiate or continue PPI therapy should be sound and PPIs should only be prescribed when there is an appropriate clinical indication. Clinical guidelines can certainly help. In this Position Paper, we have reviewed the current available guidelines, together with the systematic reviews and meta-analyses used to generate them, and synthesized the knowledge in a number of statements (i.e., Summaries of the current evidence) and in Table 3.

**Table 3:** Current indications of proton pump inhibitor (PPI) therapy

Clinical setting	PPI dose and duration
*GERD*	
Erosive Esophagitis (A/B)	Standard dose PPI therapy for 8-12 weeks
Erosive Esophagitis (C/D)	Double dose PPI therapy for 8-12 weeks
NERD	Standard dose PPI therapy for 4-8 weeks
Long-term Management (both GERD and NERD)	Standard (or half) dose PPI maintenance (continuous, intermittent or on-demand, depending on clinical characteristics of the patient)
Barrett’s Esophagus	Long-term individually-tailored PPI therapy
Extra-digestive GERD	Standard or double-dose PPI therapy for at least 12 weeks
*Eosinophilic Esophagitis*	Standard or double dose PPI therapy for 8-12 weeks
H. pylori *Eradication*	Double dose, twice daily, PPI therapy for 7-14 days (in combination with antimicrobials)
*Non* H. pylori-*related PU disease*	Standard dose PPI therapy for 4-8 weeks
*Zollinger-Ellison Syndrome*	High-dose (eventually twice daily) long-term PPI therapy
*Stress Ulcer Prophylaxis* in patients with risk factors	Standard PPI therapy by intravenous route only during ICU stay
*Dyspepsia*	
Uninvestigated Dyspepsia in Patients younger than 45 yrs	Standard or half-dose *empiric* PPI therapy for 4 weeks
Functional Dyspepsia (EPS phenotype)	Standard or half dose PPI therapy for 4-8 weeks
*NSAID-gastropathy*	
Prevention of gastro-duodenal lesions and events	Standard or half-dose PPI therapy, starting form the very first dose of NSAID in patients at GI risk
Treatment of gastro-duodenal lesions	Standard dose PPI therapy for 8 weeks
*Steroid therapy*	No need for gastroprotection unless used in combination with NSAIDs
*Anti-Platelet Therapy*	Standard dose PPI therapy, starting form the very first dose of antiplatelet agent in patients at GI risk
*Anti-Coagulant Therapy*	No need for gastroprotection unless used in combination with antiplatelet therapy
*PU Bleeding*	Intravenous bolus of 80 mg of the available injectable PPIs, followed by 8 mg/h for 72 hours
*Cirrhosis*	
Hypertensive gastropathy	No need for acid suppression
Prevention or/and treatment of esophageal ulcers after sclerotherapy or variceal band ligation	Standard dose PPI therapy for 10 days (longer treatment should be avoided taking into account the risk of spontaneous bacterial peritonitis)
*Pancreatic Diseases*	
Acute pancreatitis	No benefits from acid suppression
Chronic pancreatitis	Standard PPI therapy *only* in patients with steatorrhea, refractory to enzyme replacement therapy

Guidelines rely on both evidence and expert opinion; they are neither infallible nor a substitute for clinical judgment. They do, however, go beyond systematic reviews to recommend what should and should not be done in specific clinical circumstances. Despite guidelines being developed to improve quality of care received by patients, they have been criticized for recommending too little or too much, and even for providing reasons for national health systems or insurance payers to deny coverage [423]. Guidelines are often inflexible and can actually harm by leaving insufficient room for clinicians to tailor care to individual patient circumstances and medical history. What is best for patients overall, as recommended by guidelines, may be inappropriate for individuals [424]. Only evidence-based recommendations, which consider the balance between benefits and harms, and weigh these considerations using patient (rather than expert and societal) value, should be followed in everyday clinical practice. Taking these considerations into account, we have tried to distill the current evidence and provide physicians with clear patient-oriented recommendations, *beyond cost and reimbursement issues*. Medicine is a rapidly evolving field, however, and the validity of guidelines will not necessarily stand the test of time: indeed, today’s assumptions may no longer be valid tomorrow. Healthcare providers should therefore stay tuned and constantly update their knowledge.

PPIs continue to be prescribed outside treatment guidelines. In Europe, the most common inappropriate use of PPI is for the prevention of gastric damage in co-therapy with agents that have a low gastrotoxicity, if any, or in patients without GI risk factors for significant gastroduodenal damage [30, 31, 425–427] as well as in the prevention of stress-induced bleeding [14, 428–430]. PPIs are often started as an inpatient treatment and continued (often long-term) on discharge for non-indicated reasons [14, 431, 432].

PPIs are now available – at half the prescription dose – as over-the-counter (OTC) medications, i.e., they can be purchased directly without prescription. Although the proportion of patients taking OTC PPIs internationally is unknown, it may well represent the majority of use globally. A recent US survey [433] found that 32 % of patients with GERD symptoms were using OTC PPIs, with only 39 % of consumers using them optimally, with better symptom relief. Besides patients, 50 % of US gastroenterologists also use (i.e., suggest to patients) OTC PPIs [434]. Despite the vast majority of them (76 %) feeling that OTC brand name and generic formulations were equally effective (which is not always the case [435]), the majority of them recommended the brand medication [434].

The availability of OTC PPIs provides consumers with options other than antacids and H_2_RAs for self-medication of acid-related symptoms. Prospective clinical trials have shown the efficacy of these drugs taken on demand or intermittently on GERD management, with potential for cost reduction [17]. Although guidelines for OTC use [18, 436] suggest a short course (2 week treatment) of PPIs in patients with typical complaints (acid and/or regurgitation), and without alarm symptoms, great potential for misuse and/or overuse does exist. Major concerns include management of patients in whom symptoms persist despite acid suppression, appropriate administration, and the potential masking for more serious pathology, like malignancy. Therefore, guidelines and position statements are not just for specialists and general practitioners but should be extended to clinical and community pharmacists as well as to patients.

PPIs are among the safest class of drugs. Although concerns have been raised on their long-term safety, the preponderance of evidence does not strongly support the concerns, publicized over the last few years, and the absolute risk is probably low. Some adverse effects are plausible and predictable; others are idiosyncratic, unpredictable, and rare. Based on the quality of the existing evidence, the benefits of PPI treatment outweigh the potential risks in most patients, especially if PPI use is based on a relevant and appropriate indication [6, 44]. Conversely, patients treated without an appropriate therapeutic indication are only exposed to potential risks and the benefit-to-harm balance becomes very low. Consequently, the overall focus should be on the appropriateness of PPI therapy and on a regular assessment of the need for continued PPI treatment.

Nearly all the adverse outcomes associated with PPIs occur among patients who receive long-term therapy; minimizing the duration of treatment by periodically reviewing a patient’s need for acid-suppressive therapy could eliminate or substantially reduce the risk of adverse outcomes. Therefore, during continued long-term use, the clinical effects should always be reviewed and attempts be made to stop any therapy that may not be needed [397]. It is imperative to use the lowest dose of drug required to achieve the desired therapeutic goals. This may entail implementing discontinuation of treatment in asymptomatic patients as well as step-down [437, 438], intermittent [437, 439, 440], or on-demand PPI therapy [440–442] for maintenance of GERD. It should be emphasized, however, that PPI treatment in GERD is merely palliative in nature, since it does not address the underlying pathophysiology, something only ARS is able to achieve [443–445]. Therefore, in young fit patients needing continuous acid suppression, fundoplication should be considered. Intermittent or on-demand PPI therapy is not suitable for NSAID users, since the risk of serious GI events is constant in those patients with GI risk factors and can persist for some time after stopping therapy [170, 222].

Under the current situation of PPI misuse, opportunities do exist to increase appropriateness in order to enhance effectiveness and safety of drug therapy as well as minimize overall healthcare costs. As always, education is the key [7, 491]. Issuing guidelines and implementing them represent the most rational approach to the problem. Judicious surveillance of hospital use and prescription refills in the outpatient settings [416], with re-evaluation and justification for continued treatment, can minimize the potential for adverse effects and achieve cost saving. However, surveillance must be close and continuous since the attained benefits could be short lasting. Indeed, in a recent Canadian study [446], de-prescribing guidelines were associated with a decline in PPI use during the initial 6 months, but prescription patterns began to climb back to baseline afterwards.

## Conclusions

Overall, PPIs are irreplaceable drugs in the management of acid-related diseases. However, PPI treatment – as any kind of drug therapy – is not without risk of adverse effects. The overall benefits of therapy and improvement in quality of life significantly outweigh potential risks in most patients, but those without clear clinical indication are only exposed to the risks of PPI prescription. Adhering to evidence-based guidelines represents the only rational approach to an effective and safe PPI therapy.

## Abbreviations

ACG: American College of Gastroenterology
AIDS: acquired immunodeficiency syndrome
AIN: acute interstitial nephritis
AP: acute pancreatitis
AIGO: Italian Association of Hospital Gastroenterologists
ARS: anti-reflux surgery
CIB: clinically important bleeding
COX: cyclooxygenase
CYP 450: cytochrome P450
CP: chronic pancreatitis
CV: cardiovascular
DDIs: drug-to-drug interactions
EoE: eosinophilic esophagitis
EPS: epigastric pain syndrome
ERT: enzyme replacement therapy
FD: functional dyspepsia
FIMMG: Italian Federation of General Practitioners
GER: gastroesophageal reflux
GERD: gastroesophageal reflux disease
GI: gastrointestinal
*H. pylori*: *Helicobacter pylori*
H_2_RA: H_2_ receptor antagonist
ICU: intensive care unit
NAB: nocturnal acid breakthrough
NERD: non-erosive reflux disease
NNT: number needed to treat
NSAIDs: non-steroidal anti-inflammatory drugs
OTC: over-the-counter
OR: odds ratio
PDS: Postprandial distress syndrome
PPIs: proton pump inhibitors
PPI-REE: proton pump inhibitor-responsive esophageal eosinophilia
PU: peptic ulcer
RCT: randomized controlled trial
SIF: Italian Society of Pharmacology
SSRIs: selective serotonin reuptake inhibitors
SIBO: small intestinal bacterial overgrowth
SUP: stress ulcer prophylaxis
UGIB: upper GI bleeding
ZES: Zollinger–Ellison syndrome

## Footnotes

1 The term “coxib” is being used in the medical/scientific literature as synonymous of “selective COX-2 inhibitor”, which is not the case. The term “coxib” is a World Health Organization term used to describe a chemical class. It does not describe any pharmacological activity, nor indicate anything regarding COX-2 selectivity. Actually, there are members of the coxib family (e.g., SC-560) that selectively inhibit COX-1 [447].
